# The Type VI Secretion TssEFGK-VgrG Phage-Like Baseplate Is Recruited to the TssJLM Membrane Complex via Multiple Contacts and Serves As Assembly Platform for Tail Tube/Sheath Polymerization

**DOI:** 10.1371/journal.pgen.1005545

**Published:** 2015-10-13

**Authors:** Yannick R. Brunet, Abdelrahim Zoued, Frédéric Boyer, Badreddine Douzi, Eric Cascales

**Affiliations:** 1 Laboratoire d’Ingénierie des Systèmes Macromoléculaires, Institut de Microbiologie de la Méditerranée, Aix-Marseille Université, CNRS–UMR 7255, Marseille, France; 2 Laboratoire d’Ecologie Alpine, Université Joseph Fourier, Grenoble, France; 3 Architecture et Fonction des Macromolécules Biologiques, Aix-Marseille Université, CNRS–UMR 7257, Marseille, France; University of Geneva Medical School, SWITZERLAND

## Abstract

The Type VI secretion system (T6SS) is a widespread weapon dedicated to the delivery of toxin proteins into eukaryotic and prokaryotic cells. The 13 T6SS subunits assemble a cytoplasmic contractile structure anchored to the cell envelope by a membrane-spanning complex. This structure is evolutionarily, structurally and functionally related to the tail of contractile bacteriophages. In bacteriophages, the tail assembles onto a protein complex, referred to as the baseplate, that not only serves as a platform during assembly of the tube and sheath, but also triggers the contraction of the sheath. Although progress has been made in understanding T6SS assembly and function, the composition of the T6SS baseplate remains mostly unknown. Here, we report that six T6SS proteins–TssA, TssE, TssF, TssG, TssK and VgrG–are required for proper assembly of the T6SS tail tube, and a complex between VgrG, TssE,-F and-G could be isolated. In addition, we demonstrate that TssF and TssG share limited sequence homologies with known phage components, and we report the interaction network between these subunits and other baseplate and tail components. In agreement with the baseplate being the assembly platform for the tail, fluorescence microscopy analyses of functional GFP-TssF and TssK-GFP fusion proteins show that these proteins assemble stable and static clusters on which the sheath polymerizes. Finally, we show that recruitment of the baseplate to the apparatus requires initial positioning of the membrane complex and contacts between TssG and the inner membrane TssM protein.

## Author Summary

In the environment, bacteria compete for privileged access to nutrients or to a particular niche. Bacteria have therefore evolved mechanisms to eliminate competitors. Among them, the Type VI secretion system (T6SS) is a contractile machine functionally comparable to a crossbow: an inner tube is wrapped by a contractile structure. Upon contraction of this outer sheath, the inner tube is propelled towards the target cell and delivers anti-bacterial effectors. The tubular structure assembles on a protein complex called the baseplate. Here we define the composition of the baseplate, demonstrating that it is composed of five subunits: TssE, TssF, TssG, TssK and VgrG. We further detail the role of the TssF and TssG proteins by defining their localizations and identifying their partners. We show that, in addition to TssE and VgrG that have been shown to share homologies with the bacteriophage gp25 and gp27-gp5 proteins, the TssF and TssG proteins also have homologies with bacteriophage components. Finally, we show that this baseplate is recruited to the TssJLM membrane complex prior to the assembly of the contractile tail structure. This study allows a better understanding of the early events of the assembly pathway of this molecular weapon.

## Introduction

In the environment, bacteria endure an intense warfare. Bacteria collaborate or compete to acquire nutrients or to efficiently colonize a niche. The outcome of inter-bacteria interactions depends on several mechanisms including cooperative behaviors or antagonistic activities [1]. The newly identified Type VI secretion system (T6SS) is widely distributed among proteobacteria and has been reported to be a key player in antagonism among bacterial communities [2–4]. Although several T6SSs have been shown to be required for full virulence towards different eukaryotic cells, most T6SSs shape bacterial communities through inter-bacteria interactions [1]. In both cases, T6SSs inject toxic effectors into target/recipient cells. A number of anti-bacterial toxins have been recently identified and carry a versatile repertoire of cytotoxic activities such as peptidoglycan hydrolases, phospholipases or DNases [1,5,6]. Delivery of these toxins into the periplasm or cytoplasm of the target cell leads to a rapid lysis that usually occurs within minutes [7–9].

At a molecular level, the T6SS core apparatus is composed of 13 conserved subunits that assemble a long cytoplasmic tubular structure tethered to the cell envelope by a trans-envelope complex [3,10–12]. The composition, structure and biogenesis of the membrane-associated complex has been well characterized over the last years. It is composed of three proteins: TssL, TssM and TssJ. The TssL and TssM proteins interact in the inner membrane whereas the periplasmic domain of TssM contacts the TssJ outer membrane lipoprotein [13–15]. The current model considers the cytosolic complex of the T6SS to be similar to tails of contractile bacteriophages. These two related structures feature a cell-puncturing syringe and a contractile sheath wrapping an inner tube. The T6SS inner tube is composed of Hcp hexamers stacked on each other [16–18]. The cell-puncturing syringe assembles from a trimer of the VgrG protein tipped by the PAAR protein and is thought to cap the Hcp tube [16,19]. This structure is structurally comparable to the tail tube composed of polymerized gp19 proteins capped by the gp27-gp5 complex–or hub–in the bacteriophage T4 [20]. The TssB and TssC proteins share structural and functional similarities with the bacteriophage T4 gp18 sheath [8,21–25]. Indeed, time-lapse fluorescence microscopy experiments using a TssB-GFP fusion revealed that these structures are highly dynamic: they assemble micrometer-long tubes that sequentially extend in tens of seconds and contract in a few milliseconds [8,9,26,27]. The mechanism of contraction is thought to be similar to that of contractile bacteriophages [22–25]. Recent fluorescence microscopy assays in mixed culture evidenced that contraction of this sheath-like structure correlates with prey killing [7–9]. Based on these data and on the mechanism of bacteriophage infection, the current model proposes that the contraction of sheath-like structure propels the Hcp inner tube towards the target cell, resulting in the cell envelope puncturing and delivery of anti-bacterial toxins [3,10,28]. In tailed phages, tube and sheath polymerize on a structure called the baseplate. The bacteriophage T4 baseplate is composed of 140 polypeptide chains of at least 16 different proteins. This highly complex structure assembles from 6 wedges surrounding the central hub. Seven proteins form the baseplate wedges (gp11, gp10, gp7, gp8, gp6, gp53 and gp25) [22,29–31]. However, in other tailed bacteriophages such as P2, the baseplate is significantly less complex as it is only composed of four different subunits: gpV (the homologue of the hub) and the wedge components W (the homologue of gp25), gpJ (gp6-like) and gpI [32,33]. Based on this observation, Leiman & Shneider formulated the concept of a minimal contractile tail-like structure [22]. In the minimal contractile tail, the baseplate could be significantly “simplified” as long as it performs its main functions: controlling tube assembly, initiating sheath polymerization and triggering sheath contraction. The minimal baseplate should then conserve the central hub and three other wedge proteins: gp6, gp25 and gp53 [22]. The central hub bears the spike and acts as a threefold to sixfold adaptor connecting the tail tube. Gp25 initiates the polymerization of the sheath. Gp6 connects the wedges together maintaining the baseplate integrity during the infection process. The role of gp53 remains unclear. However, gp53 is required for gp25 to assemble onto the gp6 ring [22]. In the T6SS, the assembly of the tail-like tube and sheath must require components that will perform similar functions. With the exceptions of TssE and VgrG, which feature striking homologies to the gp25 protein and the gp27-gp5 hub complex respectively, the components that assemble the T6SS baseplate are unknown. By analogy with the morphogenesis pathway of contractile bacteriophages, we hypothesized that the assembly of the tail tube should be impaired in absence of a functional baseplate. We therefore recently developed a biochemical approach based on inter-molecular disulfide bonds to probe the assembly of the Hcp tube *in vivo*, in the cytoplasm of enteroaggregative *Escherichia coli* (EAEC) [18]. We demonstrated that Hcp hexamers stacked in a head-to-tail manner to form *bona fide* tubular structures *in vivo* [18]. More importantly, the precise stacking organization of Hcp hexamers became uncontrolled in absence of the other T6SS components. Here, using the collection of nonpolar T6SS gene deletions we provide evidence that six T6SS proteins are required for proper assembly of Hcp tubes: TssA, TssE, TssF, TssG, TssK and VgrG. The identification of TssE and VgrG, two known homologues of bacteriophage baseplate components, validates the experimental approach. We further characterize the TssF and TssG proteins. We report that these two proteins interact and stabilize each other, and make contacts with TssE, TssK and VgrG as well as with tube and sheath components. A bioinformatic analysis suggests that TssF and TssG share similarities with the J and I proteins of the bacteriophage P2 baseplate respectively. Fluorescence microscopy experiments further show that functional GFP-TssF (_sfGFP_TssF) and TssK-GFP (TssK_sfGFP_) proteins assemble into static foci near the cell envelope. The integrity of the _sfGFP_TssF foci is dependent on TssK and its proper localization requires interactions between TssF, TssG and the cytoplasmic loop of TssM. Futhermore, co-localization experiments with mCherry-labeled TssB demonstrate that _sfGFP_TssF clusters are positioned prior to sheath subunits recruitment and remain at the base of the sheath during elongation and contraction. Taken together the biochemical and cytological approaches presented in this study provide support to the role of TssE, TssF, TssG, TssK and VgrG as T6SS baseplate components and to a sequential recruitment hierarchy (membrane complex, baseplate, tail tube/sheath) during T6SS biogenesis.

## Results

### TssA, TssE, TssF, TssG, TssK and VgrG are required for proper assembly of the Hcp tube

**Fig 1:**
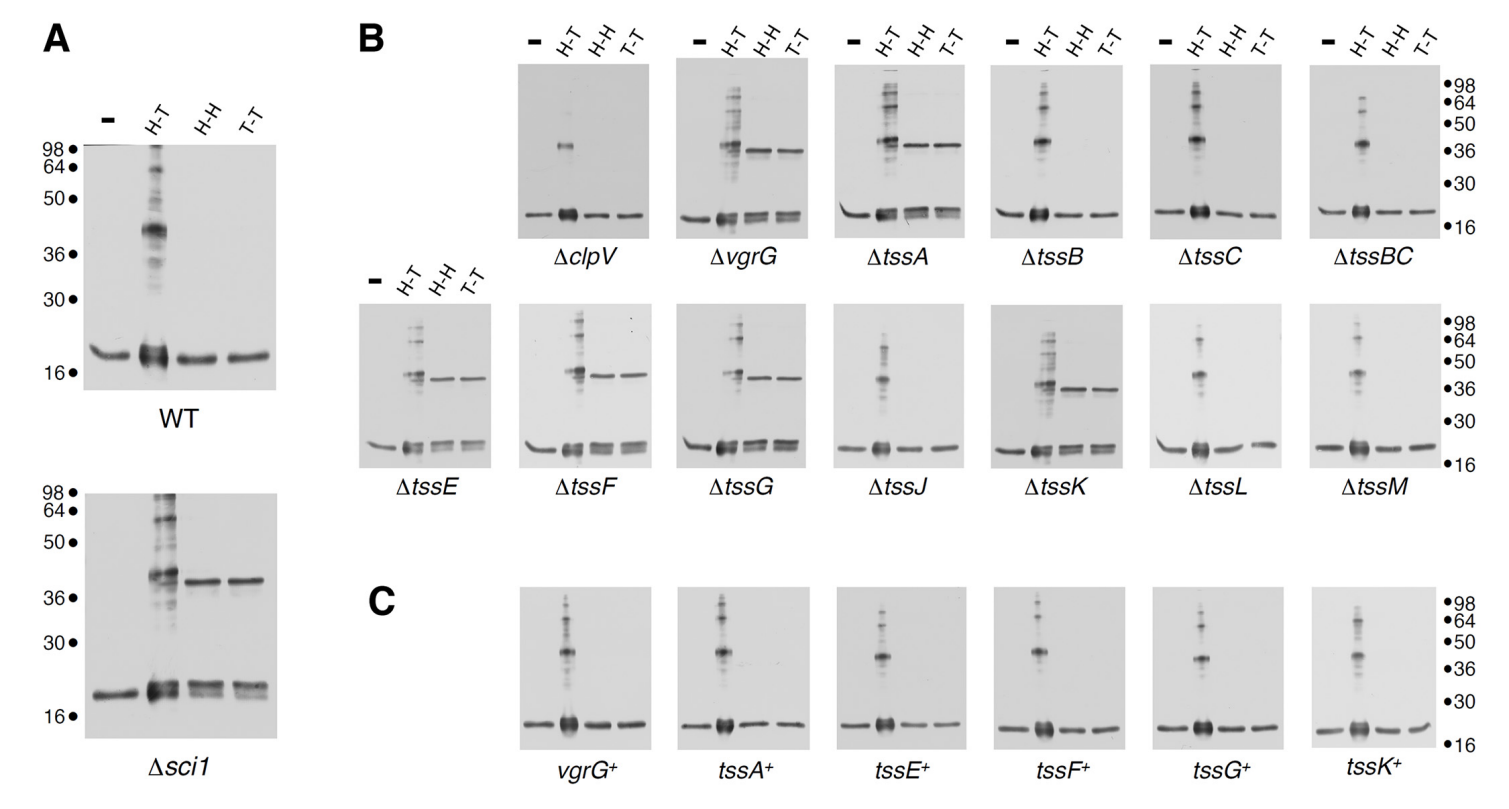
Hcp assembly defects in *vgrG*, *tssA*, *tssK*, *tssE*, *tssF* and *tssG* mutant cells. (A) Extracts from WT or Δ*sci-1* EAEC cells producing Hcp C38S (lane 1,-), Hcp C38S G96C S158C (lane 2, to probe head-to-tail packing, H-T), Hcp C38S Q24C A95C (lane 3, to probe head-to-head packing, H-H) or Hcp C38S G48C (lane 4, to probe tail-to-tail packing, T-T) after *in vivo* oxidative treatment with copper phenanthroline. (B) and (C) Extracts from EAEC Δ*tss* cells (B) or Δ*tss* cells producing the missing gene from pBAD33 complementation vectors (*tss*+) (C) and producing Hcp C38S (-), Hcp C38S G96C S158C (lane 2, H-T), Hcp C38S Q24C A95C (lane 3, H-H) or Hcp C38S G48C (lane 4, T-T) after *in vivo* oxidative treatment with copper phenanthroline. Samples were resolved on 12.5%-acrylamide SDS PAGE and Hcp and Hcp complexes were immunodetected with anti-FLAG monoclonal antibody. Molecular weight markers are indicated on the right.

**Fig 2:**
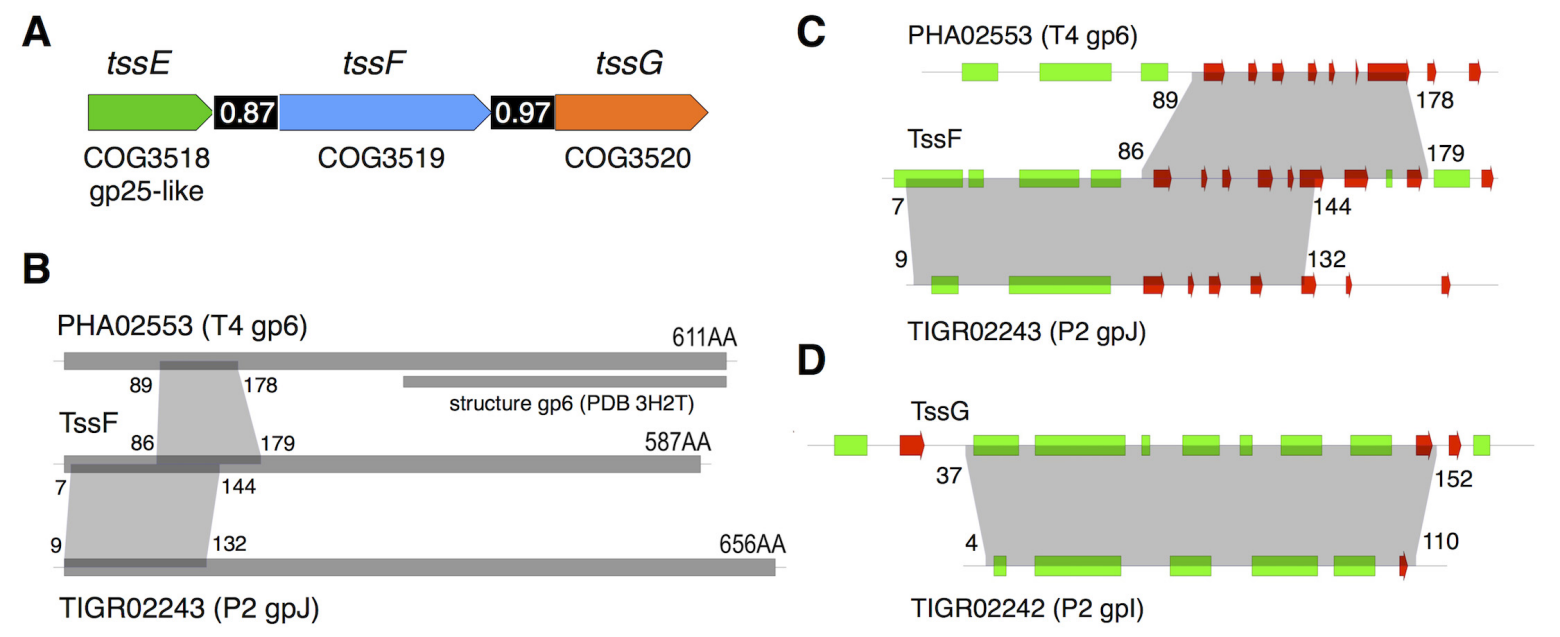
*tssE*, *tssF and tssG* genes co-occur in T6SS genes clusters and share homologies with phage baseplate components. (A) Schematic representation of the *tssE*, *tssF* and *tssG* genes with their respective COG (clusters of orthologous groups [59]) numbers. The homology between TssE and the bacteriophage T4 gp25 protein is also indicated. The number in the black bar between two genes indicates the level of co-occurrence of the two genes in bacterial genomes (adapted from [2]). (B) Schematic representations of the PHA02553 and TIGR02243 protein families (with representative members the wedge proteins gp6 of bacteriophage T4 and gpJ of bacteriophage P2 respectively) and TssF. The number of amino-acid residues for each protein is indicated, as well as the fragment of gp6 for which the crystal structure is available (PDB 3H2T, [48]). The regions of homology between the different proteins are indicated by the grey areas (amino-acid residue boundaries indicated). (C) and (D) Close-ups on the homology regions between TssF, PHA02553 and TIGR02243 and between TssG and TIGR02242 (representative member: wedge protein gpI from bacteriophage P2). The predicted secondary structures (green, -helix; red, -strand) are indicated. The grey areas indicate the regions of homology (amino-acid residue boundaries indicated).

Using an *in vivo* inter-molecular cross-linking approach, based on disulfide bond formation between adjacent cysteine residues, we recently reported that the EAEC Hcp hexamers organize head-to-tail to form tubular structures in the cytoplasm of EAEC. Importantly, we also demonstrated that these hexamers stack randomly in a strain lacking the Sci-1 T6SS subunits, resulting in head-to-tail, tail-to-tail and head-to-head configurations ([18], Fig 1A). During the morphogenesis of tailed bacteriophages, the tail-tube and-sheath structures polymerize on an assembly platform referred to as baseplate [22]. Additionally, the gp19 tail tube of bacteriophage T4 does not polymerize in absence of a fully functional baseplate [34]. By analogy, we reasoned that the aberrant assembly of Hcp hexamers *in vivo* could report a defective baseplate-like structure in the T6SS. We therefore probed the assembly of Hcp in each nonpolar Δ*tss* strain (each lacking an essential Tss subunit) using the disulfide bond assay. As proof of concept, we previously showed that the Sci-1 T6SS-associated spike protein, VgrG, is required for proper assembly of Hcp tubes [18]. Aside the cysteine-less Hcp C38S protein, three combinations were tested to probe head-to-tail (G96C-S158C), tail-to-tail (Q24C-A95C) or head-to-head (G48C) stacking. As previously reported, SDS-PAGE analyses of cytoplasmic extracts from Δ*hcp* oxidized cells producing these variants showed that the head-to-tail G96C-S158C combination leads to formation of dimers and higher molecular weight complexes while the tail-to-tail Q24C-A95C and head-to-head G48C combinations remain strictly monomeric (Fig 1A). Similar results were obtained for the *tssB*, *tssC* and *clpV* backgrounds, suggesting that tail sheath components do not regulate tail tube assembly (Fig 1B). This is in agreement with the morphogenesis pathway of contractile bacteriophages in which tail tube polymerization immediately precedes that of the sheath. Importantly, we also observed that Hcp hexamers properly assemble in the cytoplasm of *tssJ*, *tssL* and *tssM* mutants (Fig 1B). TssJ, TssL and TssM interact to form the trans-envelope complex that anchors the T6SS tail-like structure to the membranes [12–15]. Proper assembly of the tail tube structure is therefore independent of the membrane complex, a result in agreement with the different evolutionarily history of the T6SS membrane and phage complexes [2,35–37]. However, the controlled assembly of Hcp tubes was impaired in the *vgrG*, *tssA*, *tssE*, *tssF*, *tssG* and *tssK* backgrounds: Hcp hexamers interact in head-to-tail, tail-to-tail or head-to-head packing (Fig 1B). Proper Hcp assembly was restored in these different mutant strains when a wild-type allele of the missing gene was expressed from complementation vectors (Fig 1C). From these results, we concluded that six T6SS components, *i*.*e*. VgrG, TssA, TssE, TssF, TssG and TssK, increase the efficiency of tube formation *in vivo* and therefore control the assembly of Hcp tubes. TssE and VgrG share structural homologies with the gp25 protein and the gp27-gp5 complex (hub) respectively [16,19,35,38]. During the morphogenesis of the bacteriophage T4, the tail tube assembly initiates onto the baseplate only after the (gp27-gp5) hub complex has been recruited, while the gp25 subunit is required for functional baseplate assembly [39]. The observation that Hcp assembly was impaired in *vgrG* and *tssE* cells is therefore in agreement with the bacteriophage assembly pathway and further validates the initial hypothesis that Hcp tube proper polymerization depends on a baseplate-like structure. Based on these observations, we hypothesized that TssA, TssF, TssG and TssK may form, along with TssE and VgrG, a platform similar to the bacteriophage baseplate. However, while the TssE, TssK and VgrG subunits have been previously characterized [16,38,40], little information on TssA, TssF and TssG is available. The bioinformatics study published by Boyer *et al*. demonstrated a high level of co-occurrence between the *tssE* (COG3518), *tssF* (COG3519) and *tssG* (COG3520) genes. *tssE* and *tssF* are genetically linked in 87% of the T6SS gene clusters whereas the co-organization of *tssF* and *tssG* occurs in 97% of these clusters ([2], Fig 2A). As noted by Boyer and collaborators [2], co-occurrence usually reflects protein-protein interactions. Indeed, we show below that TssF and TssG are two components of the T6SS baseplate. By contrast, no co-occurrence of the *tssA* gene (COG3515) with *tssE*, *vgrG* or *hcp* was noticed in this study. Although TssA is required for Hcp tube formation, we will report elsewhere that it is not a component of the T6SS *per se* (Zoued, Durand *et al*., in preparation). We therefore focused our further work on the two uncharacterized *tssF* and *tssG* genes.

### TssF and TssG are components of the T6SS baseplate

Bioinformatic analyses: TssF and TssG are homologues to phage tail proteins. To gain further insights onto TssF and TssG we performed a bioinformatic analysis using HHPred (homology detection and structure prediction, [41]). The Sci-1 EAEC TssF (accession number: EC042_4542; gene ID: 387609963) and TssG (accession number: EC042_4543; gene ID: 387609964) protein sequences were used as baits to identify homologues in bacteriophages. HHpred analyses with TssF reported that the fragment comprising residues 7–144 (over 587) resembles region 9–132 of the phage tail-like protein TIGR02243 (PFAM04865) that has for prototype the protein J of phage P2. The segment encompassing residues 86–179 shares also secondary structures with residues 89–178 of gp6, a baseplate wedge component of bacteriophage T4 (Fig 2B and 2C). Similarly, the TssG fragment comprising residues 37–152 (over 303) resembles region 4–110 of the phage tail-like protein TIGR02242 (PFAM09684) that has for prototype the protein I of phage P2 (Fig 2D). The phage P2 baseplate is composed of four subunits: V, W, I and J [32]. Protein V is an homologue of the bacteriophage T4 gp27-gp5 complex (VgrG) [42] whereas protein W is the homologue of gp25 (TssE). Leiman & Shneider recently hypothesized that the baseplate of a minimal contractile structure assembles from a central hub (gp27-gp5 and the protein V in the bacteriophages T4 and P2 respectively) and three key wedge proteins (gp25, gp6 and gp53) in the bacteriophage T4; W, I and J proteins in the bacteriophage P2) [22]. The predicted structural homologies suggest that the N-terminal region of TssF corresponds to the N-terminal region of gp6 whereas TssG (and phage P2 protein I) corresponds to gp53. We propose therefore that the baseplate of the T6SS is composed of at least four subunits (VgrG (gp27-gp5; V), TssE (gp25; W), TssF (gp6; J) and TssG (gp53; I).

T6SS function: TssF and TssG are required for sheath polymerization and Hcp release. A set of elegant studies coupling genetic and biochemical approaches to electron microscopy imaging demonstrated that during the morphogenesis of bacteriophage particles the absence of baseplate components prevents polymerization of the inner tube and of the outer sheath [31,39,43–45]. Here, we followed the dynamic of a chromosomally-encoded TssB-mCherry fusion protein (TssB_mCh_) using time-lapse fluorescence microscopy. We observed that sheath assembly is abolished in *tssF* and *tssG* cells (S1A Fig). In agreement with the absence of sheath polymerization and contraction, western blot analyses of culture supernatants showed that *tssF* and *tssG* cells do not release Hcp in the medium (S1B Fig).

**Fig 3:**
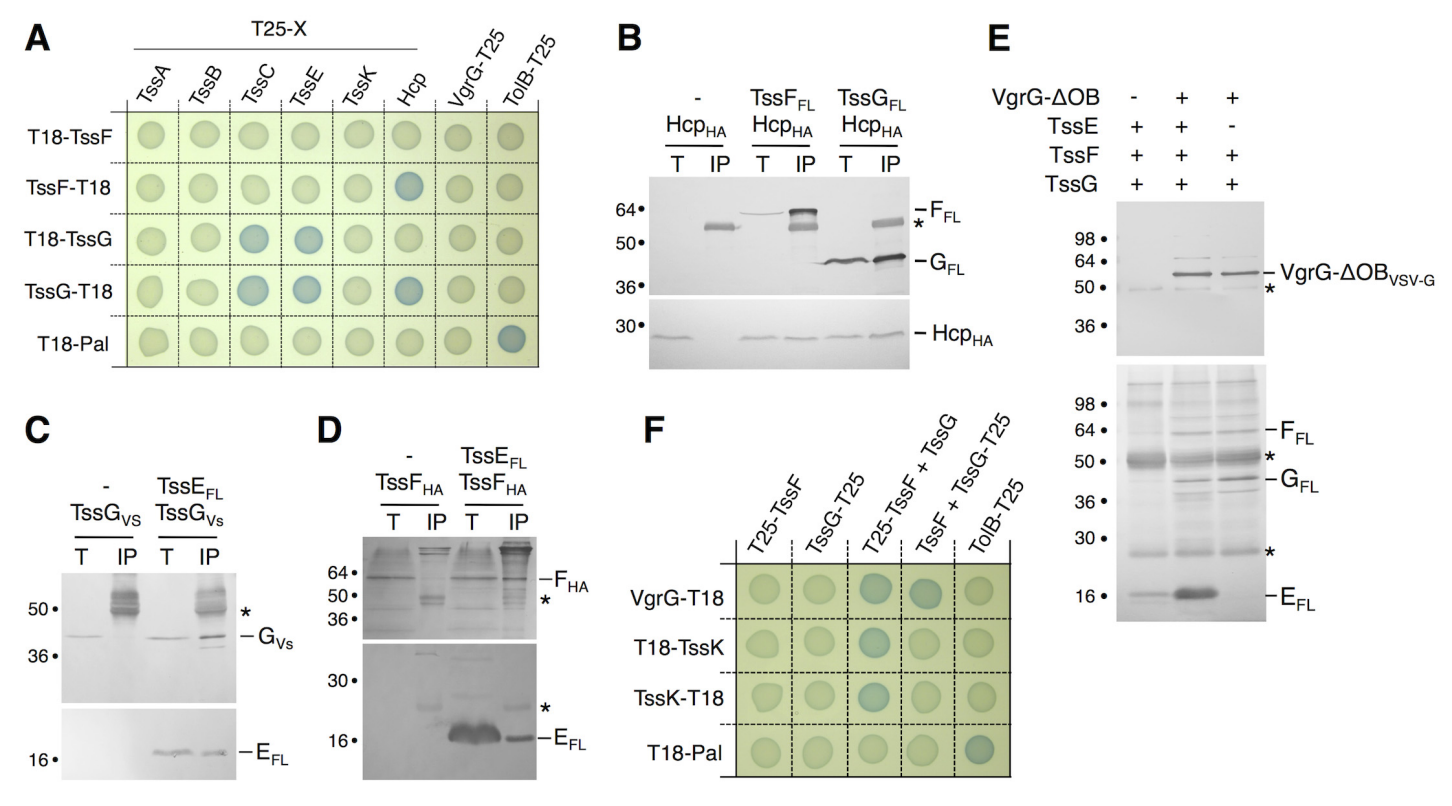
TssF and TssG interact with phage-like T6SS components. (A) Bacterial two-hybrid assay. BTH101 reporter cells producing the indicated proteins fused to the T18 or T25 domain of the *Bordetella* adenylate cyclase were spotted on plates supplemented with IPTG and the chromogenic substrate X-Gal. Interaction between the two fusion proteins is attested by the blue colour of the colony. The TolB-Pal interaction serves as a positive control. (B, C, D) Co-immunoprecipitation assays. (B) TssF and TssG interact with the tube-forming protein Hcp. Soluble extracts of *E*. *coli* K-12 W3110 strain producing FLAG-tagged TssF or TssG and HA-tagged Hcp were subjected to immunoprecipitation with anti-FLAG-coupled beads. The input (total soluble material, T) and the immunoprecipitated material (IP) were loaded on a 12.5%-acrylamide SDS-PAGE, and immunodetected with anti-FLAG and anti-HA monoclonal antibodies. Immunodetected proteins are indicated on the right. Molecular weight markers are indicated on the left. (C and D) TssG (C) and TssF (D) interact with the gp25-like subunit TssE. Soluble extracts of *E*. *coli* K-12 W3110 strain producing HA-tagged TssF and FLAG-tagged TssE or VSV-G-tagged TssG and FLAG-tagged TssE (right panel) were subjected to immunoprecipitation with anti-FLAG-coupled beads. The input (total soluble material, T) and the immunoprecipitated material (IP) were loaded on a 12.5%-acrylamide SDS-PAGE, and immunodetected with anti-FLAG, anti-HA and anti-VSV-G monoclonal antibodies. Immunodetected proteins are indicated on the right. Molecular weight markers are indicated on the left. The asterisks indicate light or heavy antibody chains. (E) Reconstitution experiments. Cleared lysates of cells producing VgrG-OB_VSVG_, TssE_FL_, TssF_FL_ or TssG_FL_ were mixed as indicated (+) and complexes were subjected to immunoprecipitation with anti-VSV-G-coupled beads. The immunoprecipitated materials were loaded on a 12.5%-acrylamide SDS-PAGE and immunodetected with anti-VSV-G (upper panel) and anti-FLAG (lower panel) monoclonal antibodies. Mixes were TssE_FL_, TssF_FL_ and TssG_FL_ alone (lane 1) or VgrG-OB_VSVG_ with TssE_FL_, TssF_FL_ and TssG_FL_ (lane 2) or with TssF_FL_ and TssG_FL_ (lane 3). The asterisks indicate light or heavy antibody chains. (F) Bacterial two-hybrid assay. BTH101 reporter cells producing the indicated proteins fused to the T18 or T25 domain of the *Bordetella* adenylate cyclase, and the third partner were spotted on plates supplemented with IPTG and the chromogenic substrate X-Gal. The TolB-Pal interaction serves as a positive control.

Interaction network: TssF and TssG interact with TssE, VgrG, TssK, Hcp and TssC. To gain further information on TssF and TssG partners, we used a bacterial two-hybrid (BTH)-based systematic approach. T18-TssF/G and TssF/G-T18 translational fusions were tested against the phage-related T6SS core-components (TssB, TssC, Hcp, TssE, VgrG and TssA) fused to the T25 domain. As shown in Fig 3A, TssF interacts with Hcp, while TssG interacts with TssC, Hcp and TssE. These pair-wise interactions were then tested by co-immunoprecipitation in the heterologous host *E*. *coli* K-12. Fig 3B shows that HA-tagged Hcp was co-immunoprecipitated with FLAG-tagged TssF or TssG. Fig 3C shows that HA-tagged TssG was co-immunoprecipitated with FLAG-tagged TssE. Interestingly, the HA-tagged TssF was also specifically co-immunoprecipitated with TssE (Fig 3D) even though the BTH assay failed to detect this interaction. Taken together, the BTH and co-immunoprecipitation assays provide evidence that TssF and TssG interact with T6SS tail components, including the TssE baseplate protein, as well as with the tube and sheath proteins Hcp and TssC. These data provide further evidence to support the Hcp assembly assay and the bioinformatics analyses to propose that TssF and TssG form with TssE and VgrG the T6SS baseplate onto which the tube (Hcp) and sheath (TssBC) polymerize.

In bacteriophages such as T4, six wedges formed by the gp6-gp25-gp53 complex assemble around the central hub. We therefore tested whether the TssE-F-G complex interacts with VgrG using a reconstitution approach. Lysates of cells producing VSV-G epitope-tagged VgrG and FLAG-tagged TssE,-F and-G were mixed prior to immunoprecipitation on anti-VSV-G resin. Fig 3E shows that VgrG efficiently precipitates TssE,-F and-G. Based on this result and on the bacterial two-hybrid approach (Fig 3A), we hypothesized that TssE links VgrG and TssF/TssG. We therefore repeated the immunoprecipitation experiments in absence of TssE. However, in these conditions we also observed that both TssF and TssG co-immunoprecipitate with VgrG (Fig 3E). Because we did not detect direct VgrG-TssF and VgrG-TssG interactions in BTH and co-immunoprecipitation assays, these results suggested that formation of a TssF-TssG complex is pre-required to interact with VgrG. Indeed, BTH experiments showed that VgrG-TssF and VgrG-TssG interactions are detected when the third partner, TssG and TssF respectively, is present (Fig 3F). A stable TssKFG complex was recently reported [46]. However, we did not observe interactions between TssK and TssF or TssG neither in this study, nor in the systematic interaction study of TssK [40]. Interestingly, further BTH experiments showed that both TssF and TssG are required to stably interact with TssK (Fig 3F), similarly to what we observed for VgrG. Therefore, we conclude that formation of the TssFG sub-complex is a pre-requisite for further interactions with VgrG and TssK.

### TssF and TssG interact and stabilize each other

**Fig 4:**
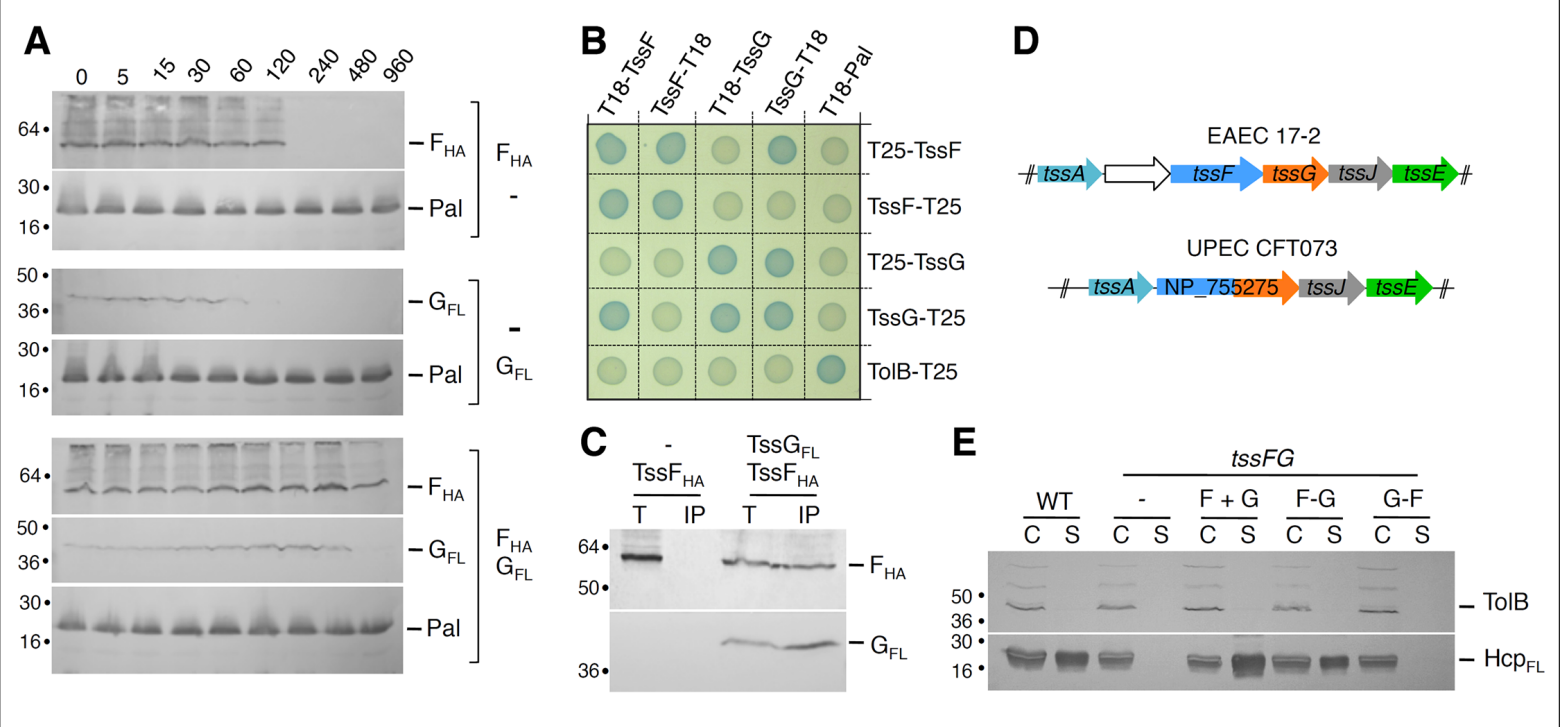
TssF and TssG interact and stabilize each other. (A) TssF and TssG are stabilized upon co-production. The steady-state levels of TssF_HA_ and TssG_FL_ proteins produced alone or together were analyzed by Western blot immunodetections of whole cells using anti-HA or anti-FLAG monoclonal antibody. The stable Pal lipoprotein was used as a loading control. Incubation periods (min) after spectinomycin/chloramphenicol treatment are indicated. (B) Bacterial two-hybrid assay. BTH101 reporter cells producing the indicated proteins fused to the T18 or T25 domain of the *Bordetella* adenylate cyclase were spotted on plates supplemented with IPTG and the chromogenic substrate Bromo-Chloro-Indolyl-β-D-galactopyrannoside. Interaction between the two fusion proteins is attested by the blue colour of the colony. The TolB-Pal interaction serves as a positive control. (C) TssF co-immunoprecipitates with TssG. Soluble extracts of *E*. *coli* K-12 W3110 strain producing TssF_HA_ and TssG_FL_ were subjected to immunoprecipitation with anti-FLAG-coupled beads. The total soluble material (T) and the immunoprecipitated material (IP) were loaded on a 12.5%-acrylamide SDS-PAGE, and immunodetected with anti-HA and anti-FLAG monoclonal antibodies. Immunodetected proteins are indicated on the right. Molecular weight markers are indicated on the left. (D) The genetic organization of the *tssF* and *tssG* genes in EAEC 17–2 and *E*. *coli* UPEC CFT073. Orthologous genes are schematically represented with the same color. A Clustal W alignment between EAEC TssF and TssG and the CFT073 NP_755275 protein is provided in S2 Fig. (E) The TssF-TssG fusion protein is functional. Hcp_FLAG_ release was assessed by separating whole cells (C) and supernatant (S) fractions from WT, *tssFG* and complemented *tssFG* cell cultures. *tssFG* cells were complemented either with a plasmid bearing *tssF* and *tssG* genes contiguously organized (F+G), the *tssF’-‘tssG* fusion (F-G) or the *tssG’-‘tssF* fusion (G-F). A total of 2×10^8^ cells and the TCA-precipitated material of the supernatant from 5×10^8^ cells were loaded on a 12.5%-acrylamide SDS-PAGE and immunodetected using the anti-FLAG monoclonal antibody (lower panel) and the anti-TolB polyclonal antibodies (control for cell integrity; upper panel).

The experiments described above and the genetic linkage of the *tssF* and *tssG* genes suggest that TssF and TssG interact. To test this hypothesis, we first performed steady-state stability experiments in *E*. *coli* K-12 cells producing TssF, TssG or both TssF and TssG. Cells were harvested at different time points after inhibition of protein synthesis, and the stabilities of TssF and TssG were estimated by Western blot. Fig 4A shows that TssF and TssG, when produced alone, are relatively unstable proteins as TssF and TssG were undetectable 120 and 60 minutes after protein synthesis inhibition respectively. However, both proteins were stabilized when co-produced and remained detectable up to 8 hours after protein synthesis arrest. This result shows that TssF and TssG stabilize each other. The co-stabilization of these two proteins is in agreement with a recent work showing that the *Serratia* TssF and TssG proteins are unstable in absence of the other [46]. To test for direct interaction, we performed BTH and co-immunoprecipitation experiments. First, the BTH assay revealed that (i) both TssF and TssG are involved in homotypic interactions suggesting that these proteins dimerize or multimerize, and (ii) that these two proteins interact (Fig 4B). However, the location of the fusion at the C-terminus of TssF or at the N-terminus of TssG causes a steric hindrance that prevents TssF-TssG complex formation. In addition, the HA epitope-tagged TssF protein was specifically co-immunoprecipitated with FLAG-tagged TssG in the heterologous T6SS^-^ host *E*. *coli* K-12 (Fig 4C) demonstrating that this interaction is not mediated by an another T6SS components. Taken together, these experiments demonstrate that TssF and TssG form a complex that stabilizes both subunits. Interestingly, within the uropathogenic *E*. *coli* (UPEC) CFT073 T6SS gene cluster, the *tssF* and *tssG* genes are fused, leading to a *tssF'-'tssG* chimeric gene (see Fig 4D). A Clustal W protein sequence alignment of the EAEC TssF and TssG proteins with the UPEC TssF-G fusion showed that the C-terminal PG residues of TssF are fused to the N-terminal MGFP residues of TssG to yield a PGMGFP motif in the fusion protein (see S2 Fig). To test whether a fusion protein might be functional in EAEC, we constructed a Δ*tssFG* mutant strain and fused the *tssF* and *tssG* genes in frame either in the native (F-G fusion) or in the opposite orientation (G-F fusion). Both fusion proteins accumulated at comparable levels. The *tssF* and *tssG* genes were also cloned contiguously, mimicking their natural genetic organization (F+G) in EAEC. As expected, the T6SS was nonfunctional in Δ*tssFG* cells as Hcp was not released in the culture supernatant, nor in the supernatant of Δ*tssFG* cells producing TssF or TssG alone. The WT phenotype was restored upon production of both TssF and TssG, or of the TssF-TssG fusion protein; however, production the TssG-TssF “inverted” fusion protein failed to complement the Δ*tssFG* mutation (Fig 4E). Taken together, these data provide evidence that TssF and TssG interact. The observation that the TssF-TssG fusion protein is functional suggests that TssF and TssG form a sub-complex with a 1:1 stoichiometry. However, by using quantitative gel staining, English *et al*. recently reported a 2:1 molar ratio for the *Serratia* TssF:G complex [46]. Although our data suggest a 1:1 stoichiometry, we cannot rule out that the TssF-G fusion protein we engineered is subjected to partial degradation. In bacteriophage T4, a 2:1 ratio has been noted for the gp6:gp53 complex [31], in agreement with the *Serratia* data [46].

### TssF and TssG are soluble cytoplasmic subunits recruited at the IM by TssM

**Fig 5:**
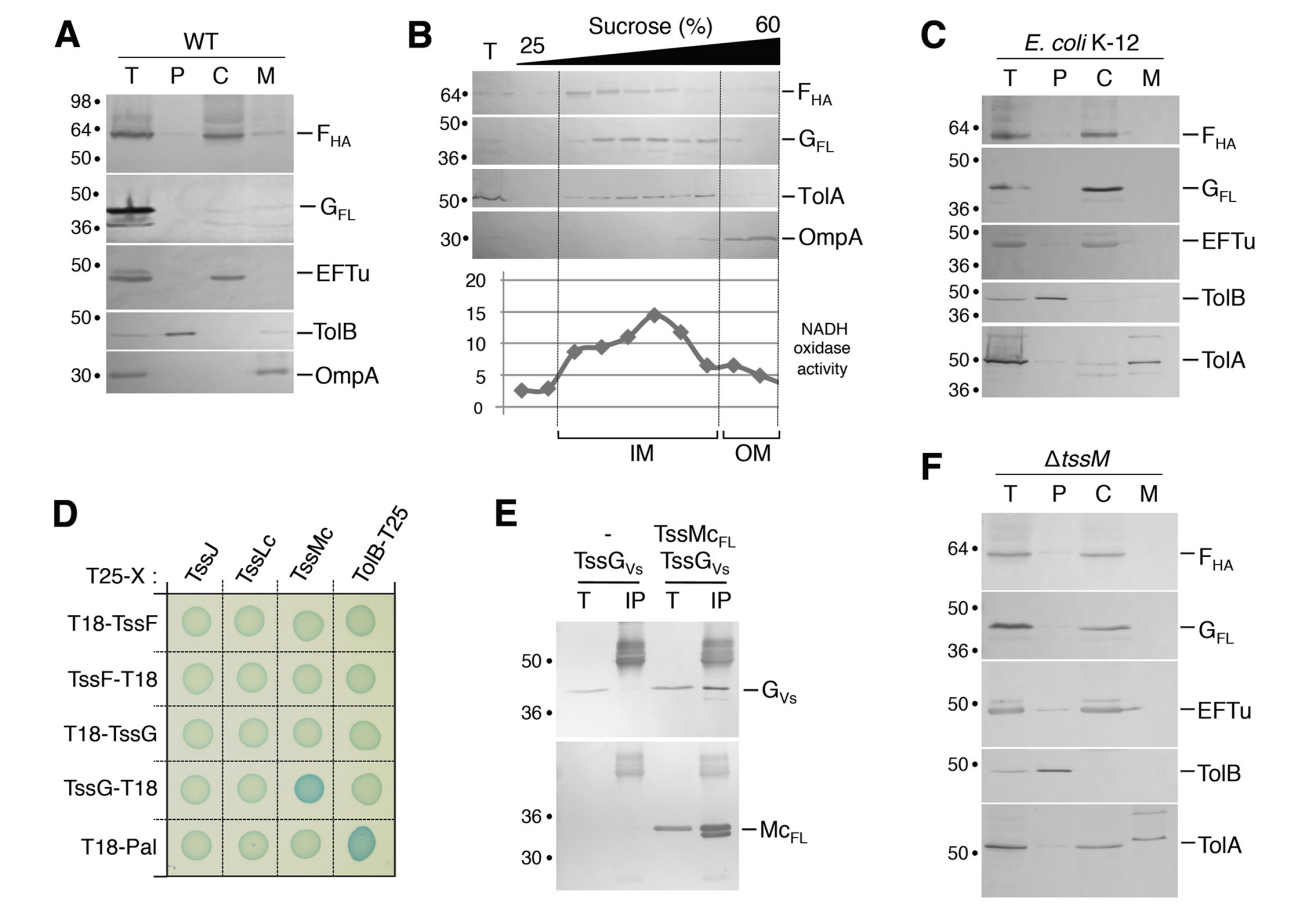
TssF and TssG are soluble proteins that associate with the IM via contacts between TssG and the cytoplasmic loop of TssM. (A) A fractionation procedure was applied to EAEC cells producing TssF_HA_ and TssG_FL_ (T, Total fraction), allowing separation between the periplasm (P), the cytoplasm (C) and membrane fractions (M). Samples were loaded on a 12.5%-acrylamide SDS-PAGE and immunodetected with antibodies directed against EFTu (cytoplasm), TolB (periplasm), OmpA (OM) proteins, and the HA epitope of TssF or the FLAG epitope of TssG. Molecular weight markers are indicated on the left. (B) Total membranes (T) from EAEC cells producing TssF_HA_ and TssG_FL_ were separated on a discontinuous sedimentation sucrose gradient. Collected fractions were analyzed for contents using the anti-TolA and anti-OmpA polyclonal antibodies or anti-HA and anti-FLAG monoclonal antibodies. In addition, NADH oxidase activity test (graph) was used as a reporter of the inner membrane protein containing fractions. NADH oxidase activity is represented relative to the total activity. The positions of the inner and outer membrane-containing fractions are indicated. (C) A fractionation procedure was applied to *E*. *coli* K-12 W3110 cells producing TssF_HA_ and TssG_FLAG_ (T, Total fraction), allowing separation between the periplasm (P), the cytoplasm (C) and membrane fractions (M). Samples were loaded on a 12.5%-acrylamide SDS-PAGE and immunodetected with antibodies directed against EFTu (cytoplasm), TolB (periplasm), TolA (IM) proteins, and the HA epitope of TssF or the FLAG epitope of TssG. Molecular weight markers are indicated on the left. (D) Bacterial two-hybrid assay. BTH101 reporter cells producing the indicated proteins fused to the T18 or T25 domain of the *Bordetella* adenylate cyclase were spotted on plates supplemented with IPTG and the chromogenic substrate Bromo-Chloro-Indolyl-β-D-galactopyrannoside. Interaction between the two fusion proteins is attested by the blue colour of the colony. The TolB-Pal interaction serves as a positive control. (E) TssG co-immunoprecipitates with the cytoplasmic domain of TssM (TssMc, amino-acids 82–360). Soluble extracts of *E*. *coli* K-12 W3110 strain producing TssG_VSVG_ only, or TssG_VSVG_ and TssMc_FL_ were subjected to immunoprecipitation with anti-FLAG-coupled beads. The total soluble material (T) and the immunoprecipitated material (IP) were loaded on a 12.5%-acrylamide SDS-PAGE and immunodetected with anti-VSVG and anti-FLAG monoclonal antibodies (additional bands correspond or heavy antibody chains). Immunodetected proteins are indicated on the right. Molecular weight markers are indicated on the left. (F) TssF and TssG remain cytoplasmic in a *tssM* deletion mutant. A fractionation procedure was applied to EAEC *tssM* cells co-producing TssF_HA_ and TssG_FL_ (T, Total fraction), allowing separation between the periplasm (P), the cytoplasm (C) and membrane fractions (M). Samples were loaded on a 12.5%-acrylamide SDS-PAGE and immunodetected with antibodies directed against EFTu (cytoplasm), TolB (periplasm), TolA (IM) proteins, and the HA epitope of TssF or the FLAG epitope of TssG. Molecular weight markers are indicated on the left.

To gain insight onto TssF and TssG, we further tested their sub-cellular localizations using cell fractionation experiments. In WT cells, both TssF and TssG mainly co-fractionated with EFTu, a cytoplasmic elongation factor (Fig 5A). Surprisingly, small but reproducible amounts of TssF and TssG were found associated with the membrane fraction (Fig 5A). TssF and TssG co-fractionate with the IM protein TolA, and the NADH oxidase activity in sedimentation density gradient experiments indicating that both proteins associate with the inner membrane (IM) (Fig 5B). These results suggest that TssF and TssG are peripherally associated with the IM, probably through protein-protein contacts. Interestingly, both proteins exclusively localized in the cytoplasmic fractions in *E*. *coli* K-12 (*i*.*e*., devoid of T6SS genes) (Fig 5C), further supporting the notion that TssF and TssG are tethered to the inner membrane by a T6SS component.

Three proteins of T6SS interact to form a membrane-associated complex that spans the cell envelope: the inner membrane TssL and TssM proteins and the outer membrane TssJ lipoprotein [12–15]. To identify TssF and TssG partners, we tested their interactions with the soluble domains of TssL, TssM and TssJ by BTH. As shown in Fig 5D, TssG interacts with the cytoplasmic loop of the inner membrane protein TssM (TssMc). This result was validated by co-immunoprecipitation: the HA-tagged TssG was co-immunoprecipitated with the FLAG-tagged TssMc domain (Fig 5E). The hypothesis that TssF and TssG were recruited to the membrane through interactions with TssM was tested by cell fractionation in Δ*tssM* cells. Fig 5F shows that in absence of TssM, TssF and TssG co-fractionate exclusively with the cytoplasmic marker EFTu and do not associate with the membrane fraction anymore. Taken together, these data show that TssF and TssG are recruited at the cytoplasmic face of the inner membrane via interactions with the cytoplasmic loop of TssM. However, association to the membrane complex might be stabilized by additional contacts involving TssFG-TssK and TssK-TssL or TssK-TssM interactions [40].

### TssF assembles stable and static clusters on which the sheath polymerizes

**Fig 6:**
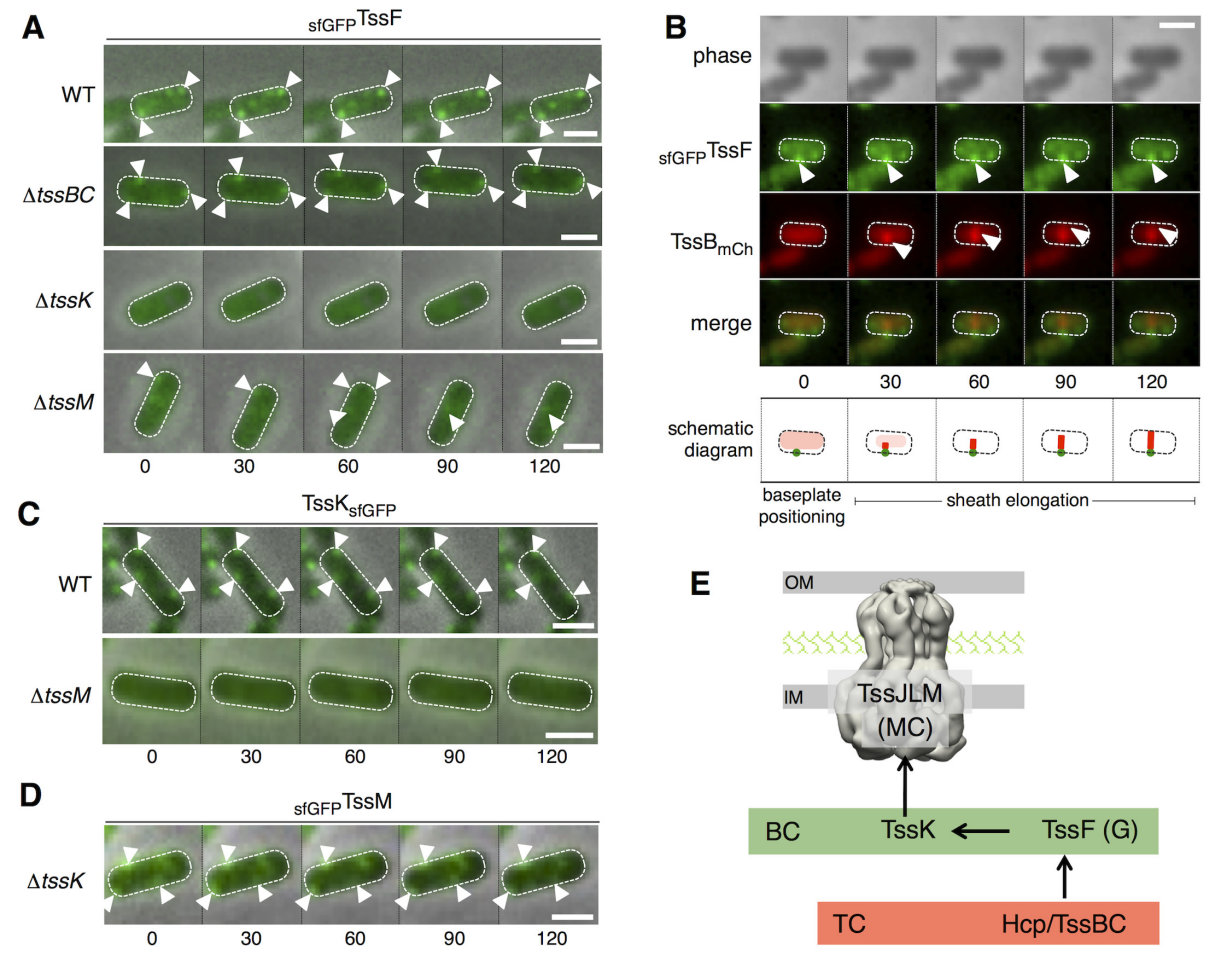
TssF and TssK assemble into static foci that serve as platform for sheath polymerization. (A) Time-lapse fluorescence microscopy recordings showing localization and dynamics of the functional _sfGFP_TssF fusion protein in wild-type (WT) cells (upper panel) or *tssBC* (second panel from top) *tssK* (third panel from top) or *tssM* (lower panel) derivatives. Individual images were taken every 30 sec. The positions of foci are indicated by white triangles. Scale bars are 1 m. Statistical analyses (number of foci/cell and dynamics) are shown in S3A and S3B Fig. (B) Sheath polymerization events initiate on _sfGFP_TssF foci. Fluorescence microscopy time-lapse recording of wild-type EAEC cells producing _sfGFP_TssF and TssB_mCh_. The GFP channel (top panel), mCherry channel (middle panel), and merge channels (lower panel) are shown. Individual images (from left to right) were taken every 30 sec. Baseplate foci and distal ends of the sheaths are indicated by white arrowheads. A schematic diagram summarizing the observed events is drawn below. The scale bar is 1 μm. Statistical (initial positioning of _sfGFP_TssF clusters, percentage of sheaths with basal _sfGFP_TssF foci) and kymograph analyses are shown in S3C and S3D and S3E Fig. (C) Time-lapse fluorescence microscopy recordings showing localization and dynamics of the functional TssK_sfGFP_ fusion protein in wild-type (WT) cells (upper panel) or its *tssM* (lower panel) derivative. Individual images were taken every 30 sec. The positions of foci are indicated by the white triangles. Scale bars are 1 m. Statistical analyses (number of foci/cell and dynamics) are shown in S3B and S3F Fig. (D) Time-lapse fluorescence microscopy recordings showing localization and dynamics of the functional _sfGFP_TssM fusion protein in *tssK* cells. Individual images were taken every 30 sec. The positions of foci are indicated by the white triangles. Scale bars are 1 m. Statistical analyses (number of foci/cell) are shown in S3G Fig. (E) Assembly pathway between selected T6SS components. The membrane complex (MC) comprising the TssJLM protein (shown with the 11.6-A electron microscopy structure) is assembled first, and is used as docking station for TssK. TssK recruits the TssF/TssG complex to the apparatus to assemble the baseplate complex (BC, green rectangle) prior to assembly of the tail complex (TC, red rectangle) comprising the Hcp inner tube and the TssBC contractile sheath-like structure.

The previous data suggest that TssE,-F,-G,-K, VgrG assemble a baseplate structure anchored to the T6SS membrane complex. Based on the knowledge on bacteriophages, we hypothesized that this baseplate serves as assembly platform for tail tube/sheath extension. To further gain information on baseplate components cellular locations, recruitment and dynamic behavior, we engineered strains producing super-folder GFP (sfGFP) fused to the N- or C-terminus of TssE, TssF, TssG and TssK. All these chromosomal constructs were introduced at the native, original loci. Only the GFP-TssF (_sfGFP_TssF) and TssK-GFP (TssK_sfGFP_) fusions were functional. In WT cells, _sfGFP_TssF forms 1–3 foci per cell, located close to the cytoplasmic side of the envelope and with a limited dynamic (Figs 6A [upper panel] and S3A and S3B). The number of _sfGFP_TssF foci and their dynamic remained unchanged in *tssBC* cells suggesting that assembly of the T6SS sheath do not impact formation of these structures (Figs 6A and S3A and S3B). By contrast, the _sfGFP_TssF fluorescence was diffuse in *tssK* cells, demonstrating that TssK is required for proper assembly of the TssF-containing baseplates (Fig 6A). The _sfGFP_TssF behavior was different in *tssM* cells. Although ~ 95% of the cells present diffuse fluorescence, _sfGFP_TssF clusters are present in ~ 5% of the cells. These clusters do not remain tightly associated to the membrane but rather display random dynamics (Fig 6A), suggesting that the membrane complexes stabilize and anchor baseplate complexes to the IM.

Fluorescence microscopy recordings of cells producing both _sfGFP_TssF and mCherry-labeled TssB (TssB_mCh_) from their native chromosomal loci further informs the assembly mechanism of the T6SS: i) _sfGFP_TssF clusters are positioned first (Figs 6B and S3C), and ii) The TssB_mCh_ sheath extends from the preassembled _sfGFP_TssF cluster (Figs 6B and S3D and S3E). In addition, the recordings show that _sfGFP_TssF foci remain associated with the sheath during all the cycle (elongation, contraction and disassembly) (S3C and S3D Fig). These observations strongly support a model in which TssF-containing complexes serve as platforms for the polymerization of the sheaths. The TssK_sfGFP_ fusion present similar behavior to _sfGFP_TssF: it assembles 1–3 stable and static foci per cell and shows diffuse localization in absence of TssM (Figs 6C and S3B and S3F). Conversely, _sfGFP_TssM forms discrete foci in WT cells [15] that assemble independently of TssK (Figs 6D and S3G). Based on these results, and in agreement with the hypothesis raised by English and co-authors [46], we suggest the TssL-M-J membrane complex serves as the docking area for the T6SS tail-like structure, the assembly of which starts with the subsequent recruitments of TssK, TssFG, Hcp and TssBC (Fig 6E).

## Discussion

**Fig 7:**
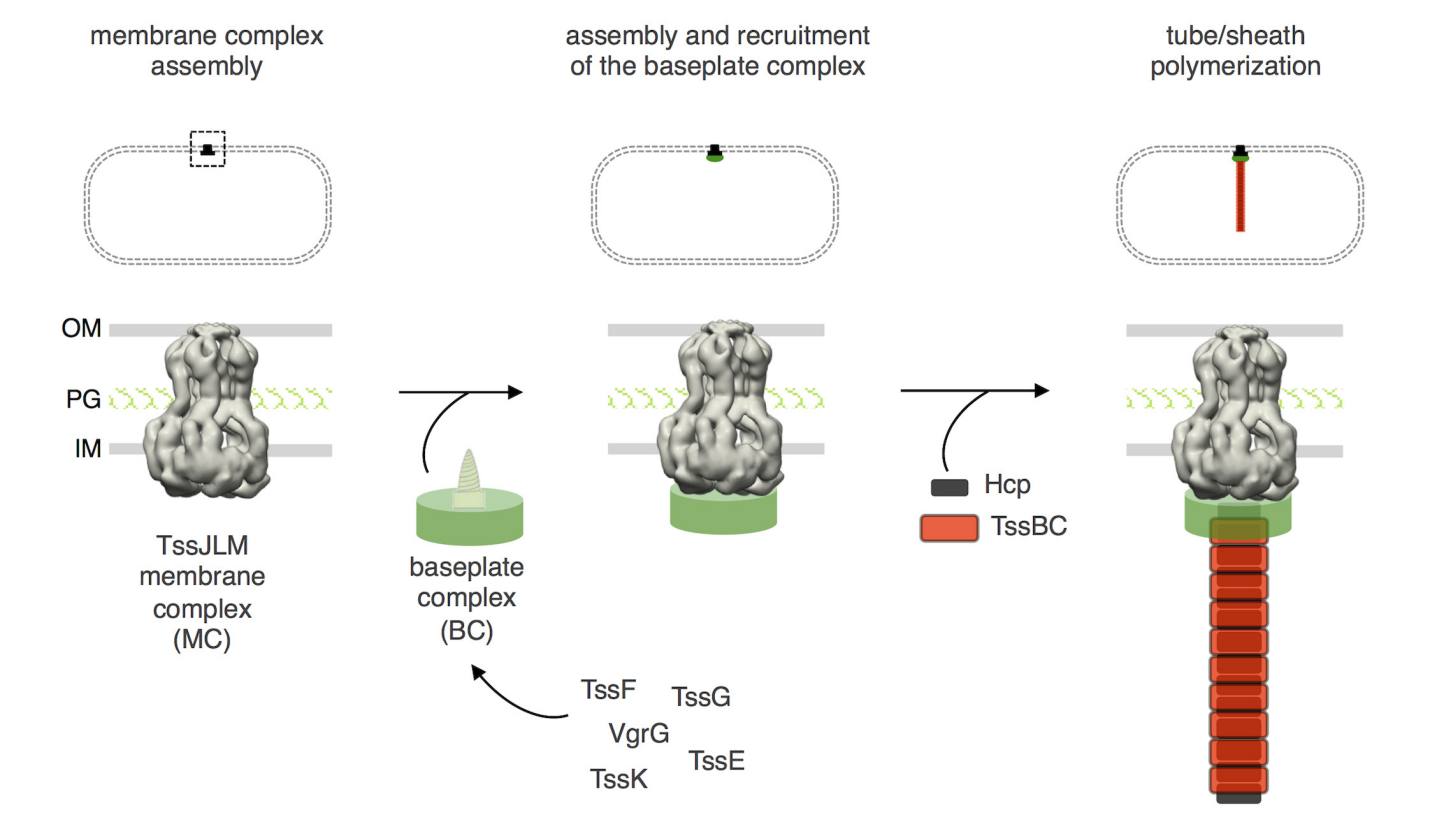
Assembly of the Type VI secretion system. Schematic representation of the different stages of T6SS biogenesis. The TssJ-L-M membrane complex is first assembled in the cell envelope (IM, inner membrane; PG, peptidoglycan; OM, outer membrane) and recruits the baseplate-like assembly platform constituted of TssE, TssF, TssG, TssK, VgrG and possibly TssA (platform represented as a green disk with the VgrG green screw). Polymerization of the tail tube (Hcp rings, black rectangles) and sheath (TssBC strands, red rectangles) is initiated after completion of the platform.

Recent studies have evidenced that the T6SS assembles a cytosolic structure similar to the tail tube and sheath of contractile bacteriophages [16,23–26]. During bacteriophage morphogenesis, the tube and sheath polymerize onto the baseplate that initiates and guides the assembly process [29–31]. In addition, the baseplate undergoes large conformational rearrangements upon landing on a target cell that ultimately trigger tail contraction [30]. In the bacteriophage T4, the baseplate is a complex structure; however, simplest baseplates exist in other contractile bacteriophages. Based on baseplates comparison, Leiman & Shneider proposed that only four components will be required to have a functional baseplate: the gp27-gp5 hub complex (or spike) and the gp25, gp6 and gp53 wedge subunits [22]. However, in the T6SS, the identity and composition of the baseplate that controls the assembly of the tube/sheath structure are not known [10–12,37]. To identify these components, we developed an assay to probe formation of Hcp tubes *in vivo* [18]. Employing systematically this assay in all T6SS gene deletion mutant strains, we identified six T6SS core components required for the controlled polymerization of the Hcp tube. This experimental approach was validated by the fact we identified VgrG and TssE, the T6SS homologues of the bacteriophage spike/hub complex and gp25 subunits respectively. Among the four remaining subunits, TssA, TssK, TssF and TssG, we showed that the two laters share limited but significant homologies with protein J and I, two baseplate components of phage P2, respectively [32,33]. In addition to bacteriophages and T6SS, homologues of P2 baseplate I and J proteins, as well as homologues of the spike/hub and gp25, are found in anti-feeding prophages, *Photorhabdus* Virulence Cassettes and R-pyocins [47] suggesting that they likely represent the core of phage-like protein translocation machineries. A schematic comparison of the homology and contacts between bacteriophage T4 and T6SS components is shown in S4 Fig. In this study, we focused our work on the TssF and TssG proteins. We demonstrated that TssF and TssG interact with each other and with TssE. Although we have not addressed the biological relevance of these interactions, these results are in agreement with the co-occurrence of these three genes in T6SS gene clusters [2], as well as with the observation that the bacteriophage T4 homologues of TssE and TssF–gp25 and gp6 –interact [48]. Interestingly, Aksyuk and coauthors showed that the gp6-gp25 interaction involves the N-terminal fragment of gp6, the fragment that shares homology with TssF [48]. In addition, contacts were detected with TssK, VgrG and components of the tube (Hcp) and of the sheath (TssC). Interestingly, contacts with TssK and VgrG require the pre-formation of the TssF-G complex. Taken together these data support the idea that the T6SS baseplate is composed of the VgrG, TssE, TssF, TssG and TssK subunits, on which the Hcp tube and the TssB-TssC sheath will sequentially polymerize (Fig 7). Indeed, co-localization studies showed that TssF is recruited to the apparatus prior to sheath extension. Based on the observations that this baseplate is docked to the membrane complex and remains at the base of the extended tail, it likely corresponds to the structure observed at the same location on T6SS electron cryo-tomographs [26].

The phage wedge and baseplate assembly pathways are regulated by the stepwise addition of the different subunits in a strict order [39,44]. Regarding T6SS biogenesis, the results presented here as well as three recent studies [15,40,46] support the proposal that the TssLMJ membrane complex is first assembled and that the T6SS is built by the hierarchical addition of TssK and TssFG. Alternatively, complete baseplates may assemble prior to docking to the membrane complex (Fig 7). Indeed, the baseplate-like structure is anchored to the inner membrane by a network of interactions including contacts between TssK and TssL, TssK and TssM, and TssG and TssM ([40] and this study). One may hypothesize that TssK is recruited to the TssJLM complex, and the interaction between the baseplate-like structure and the membrane complex is then stabilized by additional contacts between TssM and the TssFG complex. This assembly pathway is supported by fluorescence microscopy recordings demonstrating that TssF is not recruited to the apparatus in absence of TssK or TssM, and that TssK is not recruited to the apparatus in absence of TssM (Fig 6). Interestingly, in absence of TssM, TssF-containing complexes (baseplates?) are assembled–albeit at significant lower levels, suggesting that these complexes are stabilized by the membrane complex. The TssM-independent assembly of these TssF-containing complexes is in agreement with the observations that (i) T6SS membrane and baseplate/tail complexes have distinct evolutionarily histories [2] and (ii) that the membrane complex is not required for proper assembly of Hcp tubes (Fig 1C). Strikingly, phage baseplates display 6-fold symmetry [22,29] and one could assume that an identical symmetry will apply for T6SS baseplates. However, the T6SS membrane complex has been recently show to have 5-fold symmetry [15]. Understanding how a 6-fold symmetry structure successfully and functionally associates to a 5-fold symmetry docking station remains to be elucidated. Once the baseplate is docked to the TssJLM complex, the Hcp inner tube/TssBC outer sheath polymerization can proceed (Fig 7). Indeed, interaction studies showed that TssF and TssG are connected to Hcp and TssC, and might initiate tail extension. Data reporting the specific role of TssA during T6SS biogenesis will be reported elsewhere (Zoued, Durand *et al*., in preparation). The spatio-temporal recruitment of TssE and VgrG during the T6SS assembly pathway and their contributions to the structure of the T6SS baseplate are not yet known and will require further investigations. In contractile tailed bacteriophages, the baseplate serves as an assembly platform for the tube/sheath polymerization and triggers contraction of the sheath by transducing conformational changes from the fibers upon landing on host cells. It will be important to define the contribution of the T6SS baseplate to the control of T6SS sheath dynamics upon contact with prey cells.

## Materials and Methods

### Bacterial strains, medium, growth conditions and chemicals

*Escherichia coli* K-12 DH5α was used for cloning procedures, W3110 for co-immunoprecipitation, and BTH101 for the bacterial two-hybrid assay. The enteroaggregative *E*. *coli* strain 17–2 was used for this study. Strains were routinely grown in LB broth at 37°C, with aeration. For induction of the *sci-1* T6SS gene cluster, cells were grown in Sci-1-inducing medium (SIM: M9 minimal medium supplemented with glycerol (0.2%), vitamin B1 (1 μg/mL), casaminoacids (40 μg/mL), LB (10% v/v)) [49]. Plasmids and mutations were maintained by the addition of ampicillin (100 μg/ml for K-12, 200 μg/ml for EAEC), kanamycin (50 μg/ml for K-12, 50 μg/ml for chromosomal insertion on EAEC, 100 μg/ml for plasmid-bearing EAEC) or chloramphenicol (40 μg/ml). L-arabinose was purchased from Sigma-Aldrich, anhydrotetracyclin (AHT–used at 0.2 μg/ml throughout the study) from IBA. The strains, plasmids and oligonucleotides used in this study are listed in S1 Table.

### Strain construction

Δ*tss* deletion mutant strains were constructed using the modified one-step inactivation procedure [50] using red recombinase expressed from pKOBEG [51] as previously described [52]. The kanamycine cassette from pKD4 [50] was amplified with oligonucleotides carrying 50-nucleotide extensions homologous to regions adjacent to the target gene. The Polymerase Chain Reaction (PCR) product was column purified (Promega PCR and Gel Clean up) and electroporated. Kanamycin resistant clones were recovered and the insertion of the kanamycin cassette at the targeted site was verified by PCR. The kanamycin cassette was then excised using plasmid pCP20 [50], and the final strain was verified by PCR. The Δ*sci-1* deletion strain, which comprises a deletion of the *tssB-tssE* fragment (*i*.*e*., all the T6SS genes), was constructed similarly. Chromosomal fluorescent reporter insertions were obtained by the same procedure using pKD4-*gfp*, p*gfp*-KD4 and p*mCh*-pKD4 as templates for PCR amplification.

### Plasmid constructions

PCR were performed with a Biometra thermocycler, using the Pfu Turbo DNA polymerase (Stratagene; La Jolla, CA). Custom oligonucleotides were synthesized by Eurogentec. Constructions of pOK-Hcp_HA_ and pUC-Hcp_FLAG_ and its derivatives have been previously described [18,52]. pTssF-HA has been constructed by insertion of *Eco*RI-*Xho*I PCR fragment into pMS600 digested by the same enzymes. All other plasmids have been constructed by restriction-free cloning [53]: the gene of interest was amplified with oligonucleotides carrying 5’ extensions annealing to the target vector. The product of the first PCR has then been used as oligonucleotides for a second PCR using the target vector as template. All constructs have been verified by DNA sequencing (MWG).

### Hcp cross-linking assay

*In vivo* disulfide cross-linking assay was performed as previously described [18].

### Bacterial two-hybrid assay

We used the adenylate cyclase-based two-hybrid technique using previously published protocols [40,54,55]. Briefly, pairs of proteins to be tested were fused to the two catalytic domains T18 and T25 of the *Bordetella* adenylate cyclase. After co-transformation of the BTH101 strain with the two plasmids producing the fusion proteins, plates were incubated at 30°C for 2 days. 600 μl of LB medium supplemented with ampicillin, kanamycin and 0.5 mM isopropyl—thio-galactoside (IPTG) were inoculated with independent colonies. Cells were grown at 30°C overnight and spotted on LB agar plates supplemented with ampicillin, kanamycin, IPTG (0.2 mM) and 5-bromo-4-chloro-3-indolyl—D-galactopyrannoside (X-Gal), the chromogenic substrate of the -galactosidase.

### Co-immunoprecipitation assay

10^11^ exponentially growing cells producing the proteins of interest were harvested, and resuspended in Tris-HCl 20 mM (pH8.0), NaCl 100 mM supplemented with protease inhibitors (Complete, Roche) and broken by three passages at the French press (1000 psi). The total cell extract was ultracentrifuged for 45 min at 20,000 × *g* to discard unsolubilized material. Supernatants were then incubated overnight at 4°C with anti-FLAG M2 affinity beads (Sigma Aldrich) or with Protein G-Agarose beads (Roche) coupled to the anti-VSVG antibody. Beads were then washed three times with Tris-HCl 20 mM (pH8.0), NaCl 100 mM. The total extract and immunoprecipitated material were resuspended in Laemmli loading buffer prior to analyses by SDS-PAGE and immunoblotting. For reconstitution experiments, cell lysates were mixed for 30 min. at 28°C prior to immune precipitation.

### Hcp release assay, fractionation and sedimentation density gradients

Hcp release assay, Fractionation, SLS differential solubilisation, discontinuous sedimentation sucrose gradients and NADH oxidase activity measurements were performed as previously described [13].

### Time-lapse fluorescence microscopy

Overnight cultures of entero-aggregative *E*. *coli* 17–2 derivative strains were diluted 1:100 in SIM medium and grown for 6 hours to an OD_600nm_ ~ 1.0 to maximize expression of the *sci-1* T6SS gene cluster that is up-regulated in iron-depleted conditions [49]. Cells were washed in phosphate buffered saline (PBS), resuspended in PBS to an OD_600nm_ ~ 50 and spotted on a thin pad of 1.5% agarose in PBS and covered with a cover slip. Microscopy recordings and digital image processing have been performed as previously described [9,15,18,40]. For statistical analyses, fluorescent foci were automatically detected. First, noise and background were reduced using the ‘Subtract Background’ (20 pixels Rolling Ball) plugin from Fiji [56]. The sfGFP foci were automatically detected by a simple image processing: (1) create a mask of cell surface and dilate (2) detect the individual cells using the “Analyse particle” plugin of Fiji (3) sfGFP foci were identified by the “Find Maxima” process in Fiji [56]. To avoid false positive, each event was manually controlled in the original raw data. Box-and-whisker representations of the number of foci per cell were made with R software. For sub-pixel resolution tracking, fluorescent foci were detected using a local and sub-pixel resolution maxima detection algorithm and tracked over time with a specifically-developed plug-in for ImageJ [56]. The *x* and *y* coordinates were obtained for each fluorescent focus on each frame. The mean square displacement was calculated as the distance of the foci from its location at *t*_0_ at each time using R software and plotted over time. For each strain tested, the mean square displacement of at least ten individual focus trajectories was calculated. Kymographs were obtained after background fluorescence substraction and sectioning using the Kymoreslicewide plug-in under Fiji [56].

### Stability of steady-state protein levels

The protein stability was assessed as previously described [57]. Exponential growing cells producing TssF, TssG or both TssF and TssG were treated with chloramphenicol (40 μg/ml) and spectinomycin (200 μg/ml). Equivalent OD samples were harvested at 0, 5, 15, 30, 60, 120, 240, 480 and 960 minutes after protein synthesis arrest, resuspended in loading buffer prior to analyzes by SDS-PAGE and Western blot immunodetection. Bacterial density (OD_600nm_) was measured throughout the experiment to verify that no growth occurred.

### Miscellaneous

Proteins suspended in loading buffer were subjected to SDS-PAGE. For detection by immunostaining, proteins were transferred onto nitrocellulose membranes, and immunoblots were probed with primary antibodies, and goat secondary antibodies coupled to alkaline phosphatase, and developed in alkaline buffer in presence of 5-bromo-4-chloro-3-indolylphosphate and nitroblue tetrazolium. Anti-TolA,-TolB,-Pal and-OmpA polyclonal antibodies are from our laboratory collection. Anti-FLAG (Sigma-Aldrich), anti-VSV-G (Sigma-Aldrich), anti-HA (Roche), anti-EFTu (Hycult Biotech) and anti-rabbit,-mouse or-rat alkaline phosphatase-conjugated goat secondary antibodies (Beckman Coulter) have been purchased as indicated and used as recommended by the manufacturer.

## Supporting Information

S1 Table Strains, plasmids and oligonucleotides used in this study. (PDF)

S1 Fig The TssF and TssG proteins are required for Type VI secretion. Effect of the *tssF* and *tssG* mutations on T6SS sheath formation (A) and Hcp protein release (B). (A) Time-lapse fluorescence recordings of WT, *tssF* or *tssG* cells carrying the *tssB-mCherry* chromosomal fusion at the original locus. Individual images were taken every 30 sec. Assembly and contraction/disassembly events are indicated by the black and white triangles respectively. Scale bars are 1 m. (B) Hcp_FLAG_ release was assessed by separating whole cells (C) and supernatant (S) fractions from WT, *tssF* or *tssG*, and complemented *tssF* or *tssG* (*tssF* ^*+*^ or *tssG* ^*+*^) cultures. A total of 2×10^8^ cells and the TCA-precipitated material of the supernatant from 5×10^8^ cells were loaded on a 12.5%-acrylamide SDS-PAGE and immunodetected using the anti-FLAG monoclonal antibody (lower panel) and the anti-TolB polyclonal antibodies (control for cell integrity; upper panel).
(TIF)

S2 Fig ClustalW alignment of TssF and TssG with the protein NP_7555275 of *E*. *coli* CFT073. TssF and the N-terminal region of NP_7555275 share 58% of identity and 75% of similarity. TssG and the C-terminal region of NP_7555275 share 53% of identity and 82% of similarity. The region corresponding to the fusion is framed.
(TIF)

S3 Fig Statistical analyses of fluorescent subunits dynamics and behavior. Statistical analyses of _sfGFP_TssF (A), TssK_sfGFP_ (F) or _sfGFP_TssM (G) localization in the indicated strains. Shown are box-and-whisker plots of the measured number of _sfGFP_TssF, TssK _sfGFP_ or _sfGFP_TssM foci per cell for each strain with the lower and upper boundaries of the boxes corresponding to the 25% and 75% percentiles respectively. The black bold horizontal bar represents the median values for each strain and the whiskers represent the 10% and 90% percentiles. Outliers are shown as open circle. *n* indicates the number of cells analyzed per strain. (B) _sfGFP_TssF and TssK_sfGFP_ foci are stable and static. Mean square displacement (in pixel) of _sfGFP_TssF clusters in WT (blue graph) or Δ*tssBC* cells (red graph) and TssKsfGFP clusters in WT cells (black graph) were measured by sub-pixel tracking of fluorescent foci and plotted over time (in minutes). (C) _sfGFP_TssF clusters assemble prior to TssBC sheaths. Kinetics of apparition of _sfGFP_TssF clusters (green line) and dynamics of TssB_mCh_ sheaths (bars; orange: elongation, blue: elongated; purple: contraction/disassembly) plotted over time (in minutes). (D) _sfGFP_TssF remains at the base of the sheath during elongation. Kymographic analysis reporting _sfGFP_TssF (green) and TssB_mCh_ (red) positions within the cell as a function of time. (E) Percentage of sheaths (identified as elongated TssB_mCh_ structures) with basal _sfGFP_TssF clusters (identified by GFP-labeled foci), (dotted bar) in the strain producing both _sfGFP_TssF and TssB_mCh_. *n* indicates the number of cells analyzed.
(TIFF)

S4 Fig Comparison between T6SS and bacteriophage T4 subunits interactions. Schematic representation of known interactions between selected bacteriophage T4 components (top) or between T6SS components (bottom). Proteins sharing sequence or structural homologies are indicated with the same color. The bacteriophage T4 wedge components are indicated in the blue box, while the T6SS membrane complex (MC), baseplate complex (BC) and tail complex (TC) are boxed in grey, green and red respectively.
(TIF)
