# Supplementary material for: The Type VI Secretion TssEFGK-VgrG Phage-Like Baseplate Is Recruited to the TssJLM Membrane Complex via Multiple Contacts and Serves As Assembly Platform for Tail Tube/Sheath Polymerization
Source: PLoS Genet. 2015 Oct 13;11(10):e1005545. doi: 10.1371/journal.pgen.1005545 (PMC4604203; doi:10.1371/journal.pgen.1005545)
Supplement: S1 Table — (PDF) [file pgen.1005545.s001.pdf]

SUPPORTING INFORMATION

The Type VI secretion TssEFGK-VgrG phage-like baseplate is recruited to the TssJLM membrane complex
assembly platform for tail tube/sheath polymerization
via multiple contacts and serves as assembly platform for tail tube/sheath polymerization

Yannick R. Brunet, Abdelrahim Zoued, Frédéric Boyer,
Badreddine Douzi, and Eric Cascales

Supplemental Table S1. Strains, plasmids and oligonucleotides used in this study.

Strains

Strains	Description and genotype	Source
E. coli K-12		
DH5α	F-, Δ(*argF-lac*)U169, *phoA*, *supE44*, Δ(lacZ)M15, *relA*, *endA*, *thi*, *hsdR*	New England Biolabs
W3110	F-, lambda- IN(*rrnD-rrnE*)1 *rph*-1	Laboratory collection
BTH101	F-, *cya-99, araD139, galE15, galK16, rpsL1 (Str r), hsdR2, mcrA1, mcrB1.*	Karimova *et al.*, 1998
Enteroaggregative *E. coli*		
17-2	WT enteroaggregative *Escherichia coli*	Arlette Darfeuille-Michaud
17-2Δ*sci-1*	17-2 deleted of the whole *sci-1* gene cluster	Brunet *et al.*, 2014
17-2Δ*tssA*	17-2 deleted of the *tssA* gene of the *sci1* T6SS gene cluster	This study
17-2Δ*tssBC*	17-2 deleted of the *tssB* and *tssC* genes of the *sci1* T6SS gene cluster	This study
17-2Δ*tssE*	17-2 deleted of the *tssE* gene of the *sci1* T6SS gene cluster	This study
17-2Δ*tssF*	17-2 deleted of the *tssF* gene of the *sci1* T6SS gene cluster	This study
17-2Δ*tssG*	17-2 deleted of the *tsG* gene of the *sci1* T6SS gene cluster	This study
17-2Δ*tssFG*	17-2 deleted of the *tssF* and *tssG* genes of the *sci1* T6SS gene cluster	This study
17-2Δ*hcp*	17-2 deleted of the *hcp* gene of the *sci1* T6SS gene cluster	Aschtgen *et al.*, 2010
17-2Δ*vgrG*	17-2 deleted of the *vgrG* gene of the *sci1* T6SS gene cluster	Brunet *et al.*, 2014
17-2Δ*tssJ*	17-2 deleted of the *tssJ* gene of the *sci1* T6SS gene cluster	Aschtgen *et al.*, 2008
17-2Δ*tssK*	17-2 deleted of the *tssK* gene of the *sci1* T6SS gene cluster	Zoued *et al.*, 2013

17-2Δ*tssL*	17-2 deleted of the *tssL* gene of the *sci1* T6SS gene cluster	Aschtgen *et al.*, 2010
17-2Δ*tssM*	17-2 deleted of the *tssM* gene of the *sci1* T6SS gene cluster	Aschtgen *et al.*, 2010
17-2Δ*clpV*	17-2 deleted of the *clpV* gene of the *sci1* T6SS gene cluster	Aschtgen *et al.*, 2008
17-2 *tssB-mCh*	*mCherry* inserted upstream the stop codon of *tssB* in 17-2	This study
17-2Δ*tssF tssB-mCh*	*mCherry* inserted upstream the stop codon of *tssB* in 17-2Δ*tssF*	This study
17-2Δ*tssG tssB-mCh*	*mCherry* inserted upstream the stop codon of *tssB* in 17-2Δ*tssG*	This study
17-2 *gfp-tssF*	*gfp-mut2* inserted downstream the start codon of *tssF* in 17-2	This study
17-2Δ*tssBC gfp-tssF*	17-2 *gfp-tssF* deleted of the *tssB* and *tssC* genes	This study
17-2Δ*tssK gfp-tssF*	17-2 *gfp-tssF* deleted of the *tssK* gene	This study
17-2Δ*tssM gfp-tssF*	17-2 *gfp-tssF* deleted of the *tssM* gene	This study
17-2 *tssF-gfp tssB-mCh*	*mCherry* inserted upstream the stop codon of *tssB* in 17-2 *gfp-tssF*	This study
17-2 *tssK-gfp*	*gfp-mut2* inserted upstream the stop codon of *tssK* in 17-2	This study
17-2Δ*tssM tssK-gfp*	17-2 *tssK-gfp* deleted of the *tssM* gene	This study
17-2 *gfp-tssM*	*gfp-mut2* inserted downstream the start codon of *tssM* in 17-2	Durand, Nguyen, Zoued *et al.*, 2015
17-2Δ*tssK gfp-tssM*	17-2 *gfp-tssM* deleted of the *tssK* gene	This study

Plasmids

Vectors	Description	Source
Expression vectors		
pOK12	cloning vector, P15A origin, P*lac*, Kan^R	Vieira & Messing, 1991
pOK-$TssF_{HA}$	*sci1 tssF* cloned into pOK12, C-terminal Hemagglutinin epitope	This study
pOK-Hcp_{HA}	*sci1 hcp* cloned into pOK12, C-terminal Hemagglutinin epitope	Aschtgen *et al.*, 2010
pUC12	cloning vector, ColE1 origin, P*lac*, Amp^R	Vieira & Messing, 1991
pUC-Hcp_{FL}	*sci1 hcp* cloned into pUC12, P*lac*, C-terminal FLAG epitope	Aschtgen *et al.*, 2008
pUC-$HcpC38S_{FL}$	Cys38-to-Ser mutation inserted into pUC-Hcp_{FL}	Brunet *et al.*, 2014
pUC-HcpC38S-Q24C-$A95C_{FL}$	Gln24-to-Cys and Ala95-to-Cys mutations inserted into pUC-$HcpC38S_{FL}$	Brunet *et al.*, 2014
pUC-HcpC38S-G96C-S158C $_{FL}$	Gly96-to-Cys and Ser158-to-Cys mutations inserted into pUC-$HcpC38S_{FL}$	Brunet *et al.*, 2014
pUC-HcpC38S-$G48C_{FL}$	Gly48-to-Cys mutation inserted into pUC-$HcpC38S_{FL}$	Brunet *et al.*, 2014
pBAD33	cloning vector, pACYC184 origin, P*ara*, *araC*, Cm^R	Guzman *et al.*, 1995
pBAD33-$TssF_{FL}$	*sci1 tssF* cloned into pBAD33, C-terminal FLAG epitope	This study
pBAD33-$TssG_{FL}$	*sci1 tssG* cloned into pBAD33, C-terminal FLAG epitope	This study
pBAD33-$TssG_{VSV-G}$	*sci1 tssG* cloned into pBAD33, C-terminal VSV-G epitope	This study
pBAD33-$VgrG_{VSV-G}$	*sci1 vgrG* cloned into pBAD33, C-terminal VSV-G epitope	This study
pBAD33-VgrGΔOB_{VSV-G}	*sci1 vgrG* deleted of the OB domain ($VgrG_{1-573}$ fragment), C-terminal VSV-G epitope	This study
pBAD33-$TssA_{VSV-G}$	*sci1 tssA* cloned into pBAD33, C-terminal VSV-G epitope	This study
pBAD33-$TssE_{VSV-G}$	*sci1 tssE* cloned into pBAD33, C-terminal VSV-G epitope	This study
pBAD33-$TssK_{VSV-G}$	*sci1 tssK* cloned into pBAD33, C-terminal VSV-G epitope	This study
pASK-IBA37	cloning vector, P*tet*, Amp^R	IBA Technology
pASK-IBA37-$TssF_{FL}$	*sci1 tssF* cloned into pASK-IBA37, C-terminal FLAG epitope	This study
pASK-IBA37-$TssG_{FL}$	*sci1 tssG* cloned into pASK-IBA37, C-terminal FLAG epitope	This study
pASK-IBA37-TssF+$TssG_{FL}$	*sci1 tssF* and *tssG* cloned into pASK-IBA37, TssG C-terminal FLAG epitope	This study
pASK-IBA37-$TssFG_{FL}$	*sci1 tssF-tssG* fusion cloned into pASK-IBA37, C-terminal FLAG epitope	This study

pASK-IBA37-TssGF$_{FL}$	*sci1 tssG-tssF* fusion cloned into pASK-IBA37, C-terminal FLAG epitope	This study
pASK-IBA37-TssE$_{FL}$	*sci1 tssE* cloned into pASK-IBA37, C-terminal FLAG epitope	This study
pASK-IBA37-TssMc$_{FL}$	*sci1 tssM* cytoplasmic region (TssM$_{62-360}$ fragment) cloned into pASK-IBA37, C-terminal FLAG epitope	This study
Bacterial Two-Hybrid vectors		
pT18-FLAG	Bacterial Two Hybrid vector, ColE1 origin, P*lac*, T18 fragment of *Bordetella pertussis* CyaA, Amp^{R}	Battesti & Bouveret, 2008
pT18-TssF	*tssF* cloned upstream the T18 coding sequence in pT18-FLAG	Zoued *et al.*, 2013
pTssF-T18	*tssF* cloned downstream the T18 coding sequence in pT18-FLAG	Zoued *et al.*, 2013
pT18-TssG	*tssG* cloned upstream the T18 coding sequence in pT18-FLAG	Zoued *et al.*, 2013
pTssG-T18	*tssG* cloned downstream the T18 coding sequence in pT18-FLAG	Zoued *et al.*, 2013
pT18-TssF	*tssF* cloned upstream the T18 coding sequence in pT18-FLAG	Zoued *et al.*, 2013
pTssF-T18	*tssF* cloned downstream the T18 coding sequence in pT18-FLAG	Zoued *et al.*, 2013
pT18-TssF+TssG	*tssF* and *tssG* cloned downstream the T18 coding sequence in pT18-FLAG	This study
pTssF+TssG-T18	*tssF* and *tssG* cloned upstream the T18 coding sequence in pT18-FLAG	This study
pT18-Pal	*pal* cloned downstream the T18 coding sequence in pT18-FLAG	Battesti & Bouveret, 2008
pT25-FLAG	Bacterial Two Hybrid vector, p15A origin, P*lac*, T25 fragment of *Bordetella pertussis* CyaA, Kan^{R}	Battesti & Bouveret, 2008
pT25-TssA	*tssA* cloned upstream the T25 coding sequence in pT25-FLAG	Zoued *et al.*, 2013
pTssA-T25	*tssA* cloned downstream the T25 coding sequence in pT25-FLAG	Zoued *et al.*, 2013
pT25-TssB	*tssB* cloned upstream the T25 coding sequence in pT25-FLAG	Zoued *et al.*, 2013
pTssB-T25	*tssB* cloned downstream the T25 coding sequence in pT25-FLAG	Zoued *et al.*, 2013
pT25-TssC	*tssC* cloned upstream the T25 coding sequence in pT25-FLAG	Zoued *et al.*, 2013
pTssC-T25	*tssC* cloned downstream the T25 coding sequence in pT25-FLAG	Zoued *et al.*, 2013
pT25-TssE	*tssE* cloned upstream the T25 coding sequence in pT25-FLAG	Zoued *et al.*, 2013
pTssE-T25	*tssE* cloned downstream the T25 coding sequence in pT25-FLAG	Zoued *et al.*, 2013
pT25-TssF	*tssF* cloned upstream the T25 coding sequence in pT25-FLAG	Zoued *et al.*, 2013
pTssF-T25	*tssF* cloned downstream the T25 coding sequence in pT25-FLAG	Zoued *et al.*, 2013
pT25-TssG	*tssG* cloned upstream the T25 coding sequence in pT25-FLAG	Zoued *et al.*, 2013
pTssG-T25	*tssG* cloned downstream the T25 coding sequence in pT25-FLAG	Zoued *et al.*, 2013

pT25-TssF+TssG	*tssF* and *tssG* cloned downstream the T25 coding sequence in pT25-FLAG	This study
pTssF+TssG-T25	*tssF* and *tssG* cloned upstream the T25 coding sequence in pT25-FLAG	This study
pT25-Hcp	*hcp* cloned upstream the T25 coding sequence in pT25-FLAG	Zoued *et al.*, 2013
pHcp-T25	*hcp* cloned downstream the T25 coding sequence in pT25-FLAG	Zoued *et al.*, 2013
pT25-VgrG	*vgrG* cloned upstream the T25 coding sequence in pT25-FLAG	Zoued *et al.*, 2013
pVgrG-T25	*vgrG* cloned downstream the T25 coding sequence in pT25-FLAG	Zoued *et al.*, 2013
pT25-$TssJ_{sol}$	$tssJ_{sol}$ ($TssJ_{2-152}$ fragment of the processed form) cloned upstream the T25 coding sequence in pT25-FLAG	Zoued *et al.*, 2013
p$TssJ_{sol}$-T25	$tssJ_{sol}$ ($TssJ_{2-152}$ fragment of the processed form) cloned downstream the T25 coding sequence in pT25-FLAG	Zoued *et al.*, 2013
pT25-TssK	*tssK* cloned upstream the T25 coding sequence in pT25-FLAG	Zoued *et al.*, 2013
pTssK-T25	*tssK* cloned downstream the T25 coding sequence in pT25-FLAG	Zoued *et al.*, 2013
pT25-TssLc	Cytoplasmic region of *tssL* ($TssL_{1-184}$ fragment) cloned upstream the T25 coding sequence in pT25-FLAG	Durand *et al.*, 2012
pTssLc-T25	Cytoplasmic region of *tssL* ($TssL_{1-184}$ fragment) cloned downstream the T25 coding sequence in pT25-FLAG	Durand *et al.*, 2012
pT25-TssMc	Cytoplasmic region of *tssM* ($TssM_{62-360}$ fragment) cloned upstream T25 in pT25-FLAG	Zoued *et al.*, 2013
pTssMc-T25	Cytoplasmic region of *tssM* ($TssM_{62-360}$ fragment) cloned downstream T25 in pT25-FLAG	Zoued *et al.*, 2013
pTolB-T25	*tolB* cloned upstream the T25 coding sequence in pT25-FLAG	Battesti & Bouveret, 2008
Vectors for chromosomal insertions		
pKD4	One-step gene inactivation vector, Kan^R	Datsenko & Wanner, 2000
pKD4-*gfp*	superfolder-*gfp* cloned downstream the 3'-FRT site of pKD4. Used for chromosomal N-terminal GFP fusion	This study
p*gfp*-KD4	superfolder-*gfp* cloned upstream the 5'-FRT site of pKD4. Used for chromosomal C-terminal GFP fusion	This study
p*m-Ch*-KD4	*mCherry* cloned upstream the 5'-FRT site of pKD4. Used for chromosomal C-terminal mCherry fusion	This study

Oligonucleotides

Name	Destination	Sequence (5’ ➔ 3’)
For strain construction [a]		
5–Δ*tssA*	deletion of the *tssA* gene	TTACAGGCAATGACGAAGATGCCGGGGATACCGTGGAGGAGACTGACTGATGTGTAGGCTGGAGCTGCTTCG
3-Δ*tssA*	deletion of the *tssA* gene	TGACTATATCCTGAAATGTTTTGTCCTGAAGATAATGCATGACAGACATCCATATGAATATCCTCCTTAGTTCC
5–Δ*tssBC*	deletion of the *tssB* and *tssC* genes	GCATCTGCGGTGATGGAACCCCTGAGATGCAGGTTTCACAGGAGAGAGCCGTGTAGGCTGGAGCTGCTTCG
3-Δ*tssBC*	deletion of the *tssB* and *tssC* genes	GCCCCGTCTTCCCATAATGGGCGATAAATCTTCATTTCCCGACACCTGCCGCATATGAATATCCTCCTTAGTTCC
5–Δ*tssE*	deletion of the *tssE* gene	TATTGAAGTATCCGGTAACACCCTGACCCTGTTACCGGTGAAGGATAAATATGTGTAGGCTGGAGCTGCTTCG
3-Δ*tssE*	deletion of the *tssE* gene	CATATATCCATCGCTGTTTATTTATTTCTGTTCTGCCCTCCCTGCTCCGGCCATATGAATATCCTCCTTAGTTCC
5–Δ*tssF*	deletion of the *tssF* gene	GAAGTCAGAGCGCGAGTCTCCGGAACAGTTAGCTTTATTCTGAGAAGATATGTGTAGGCTGGAGCTGCTTCG
3-Δ*tssF*	deletion of the *tssF* gene	ACAGAAGCGGTAAAAATTAATATACGGAAGCTGTTTCCGGTAACGGGTAACATATGAATATCCTCCTTAGTTCC
5–Δ*tssG*	deletion of the *tssG* gene	TTACCCGTTACCGGAAACAGCTTCCGTATATTAATTTTTACCGCTTCTGTTGTGTAGGCTGGAGCTGCTTCG
3-Δ*tssG*	deletion of the *tssG* gene	AACGCAATAATCAGACCGTAACCAGCCTTACCAGCGATAATCGCCATTTTCATATGAATATCCTCCTTAGTTCC
5–Δ*tssFG*	deletion of the *tssF* and *tssG* genes	GAAGTCAGAGCGCGAGTCTCCGGAACAGTTAGCTTTATTCTGAGAAGATATGTGTAGGCTGGAGCTGCTTCG
3-Δ*tssFG*	deletion of the *tssF* and *tssG* genes	AACGCAATAATCAGACCGTAACCAGCCTTACCAGCGATAATCGCCATTTTCATATGAATATCCTCCTTAGTTCC

5–Δ*tssK*	deletion of the *tssK* gene	CAGTGGTTTCGCAGATGCCGAAGGCAAAAGCGTAAGGCAGGTGTCGGGA ATGTGTAGGCTGGAGCTGCTTCG
3-Δ*tssK*	deletion of the *tssK* gene	CAGCCGGGATAAAAAATCTGTTCAGCCCGGGAGATAACAGGTTTATTCAT CATATGAATATCCTCCTTAGTTCC
5–Δ*tssM*	deletion of the *tssM* gene	GAGAAGAACATTTTATCAGTACTGTTACATCAGGAAACCAGAATGAATAA TGTGTAGGCTGGAGCTGCTTCG
3-Δ*tssM*	deletion of the *tssM* gene	CCCGGGGTGTCGTCTGGCATGCACTGAGGAGCGAATGAATGGAAGCCATC CATATGAATATCCTCCTTAGTTCC
5-*tssB-mCherry*	insertion of *mCherry* at the 3' end of *tssB*	CCGGCACTGAGTCAGACGCTGCGTGATGAACTGCGTGCACTGGTGCCGGA AAAGGCGGCAGCGGCCGGCGGAGGG
3-*tssB-mCherry*	insertion of *mCherry* at the 3' end of *tssB*	GCAACGTTCTTTTCTTTCTGTACAGACATCAGCATTTTCTCTCGTAATCCGT TAAACATATGAATATCCTCCTTAGTTCCTATTCCGAAGTTCC
5-*gfp-tssF*	insertion of *gfp-mut2* at the 5' of *tssF*	GGCATGGCTGAAGTCAGAGCGCGAGTCTCCGGAACAGTTAGCTTTATTCT GAGAAGATACGATTGTGTAGGCTGGAGCTGCTTCGAAGTTCCTATAC
3-*gfp-tssF*	insertion of *gfp-mut2* at the 5' of *tssF*	CTTTCCGGCTTCACGCAGATAACGCATTTCAGCGTCAAAATAACGCAGGG TTAAATCGTCCCCTCCGCCGGCCGCTGC
5-*tssK-gfp*	insertion of *gfp-mut2* at the 3' of *tssK*	TTCTACACCCCGGCATCGCTGGGAGATGTGAAACTGGAACTTTTTGCGGTG CTGCGGACAGCAGCGGCCGGCGGAGGG
3-*tssK-gfp*	insertion of *gfp-mut2* at the 3' of *tssK*	GGCTGACCATCAGCCAGCCGGGATAAAAAATCTGTTCAGCCCGGGAGATA ACAGGTTTATTCATATGAATATCCTCCTTAGTTCCTATTCCGAAGTTCC
5-*gfp-tssM*	insertion of *gfp-mut2* at the 5' of *tssM*	TTCTCATCCGGAGAAGAACATTTTATCAGTACTGTTACATCAGGAAACCAG AATGAATAACGATTGTGTAGGCTGGAGCTGCTTCGAAGTTCCTATAC
3-*gfp-tssM*	insertion of *gfp-mut2* at the 5' of *tssM*	CACACCAATAAATACAATCCCCGGTCGCCCAAAGCGACCAGACAGACAGG CCAGTTTATTCCCTCCGCCGGCCGCTGC

For plasmid construction [b,c,d]

IBA37-TssF$_{FL}$-5	GACAAAAATCTAGAAATAATTTTGTTTAACTTTAAGAAGGAGATATACAAATGGACGATTTAAC CCTGCGTTATTTTGACGCTG
IBA37-TssF-FLAG-3	GATGGTGATGGTGATGCGATCCTCTGCTAGCTTA**TTTATCATCGTCGTCTTTATAATC**ACCGGG CAGGCGCGGATTGTG
IBA37-TssG-FLAG-5	GACAAAAATCTAGAAATAATTTTGTTTAACTTTAAGAAGGAGATATACAAATGTTGCTGGAACA

	AAGCCAGCCAGACCAG
IBA37-TssG-FLAG-3	GATGGTGATGGTGATGCGATCCTCTGCTAGC**TTATTTATCATCGTCGTCTTTATAATC**CCAGCG ATAATCGCCATTTTCCTGAGTCTCC
IBA37-TssFG-FLAG-5	CACAATCCGCGCCTGCCCGGTGGGGGCGGATTGCTGGAACAAA GCCAGCCAGACCAG
IBA37-TssFG-FLAG-3	GATGGTGATGGTGATGCGATCCTCTGCTAGCTTA**TTTATCATCGTCGTCTTTATAATC**CCAGCG ATAATCGCCATTTTCCTGAGTCTCC
IBA37-TssGF-FLAG-5	GACAAAAATCTAGAAATAATTTTGTTTAACTTTAAGAAGGAGATATACAAATGTTGCTGGAACA AAGCCAGCCAGACCAG
IBA37-TssGF-FLAG-3	CAGCGTCAAAATAACGCAGGGTTAAATCGTCTCCGCCCCCCCAGCGATAATCGCCATTTTCCTGA GTCTCC
IBA37-TssE-FLAG-5	GACAAAAATCTAGAAATAATTTTGTTTAACTTTAAGAAGGAGATATACAAATGGTGATGCCGCG TCCTTCCCT
IBA37-TssE-FLAG-3	GATGGTGATGGTGATGCGATCCTCTGCTAGCTTA**TTTATCATCGTCGTCTTTATAATC**CGTCTG CACGTAGCGCTGCTG
IBA37-TssMc-FLAG-5	GACAAAAATCTAGAAATAATTTTGTTTAACTTTAAGAAGGAGATATACAAATGGTGATGAAAAA ATATGTTCAGGAACTGACATATC
IBA37-TssMc-FLAG-3	GATGGTGATGGTGATGCGATCCTCTGCTAGCTTA**CTTGTCATCGTCGTCCTTATAATC**TCTGCG CCAGTTATGACCTGTGCGGGA
pOK-TssF-HA-5	GTCG*GAATTC*GACGATTTAACCCTGCGTTAAAAAGACG
pOK-TssF-HA-3	CTGC*CTCGAG*ACCGGGCAGGCGCGGATTG
BAD33-TssF-5	CTCTCTACTGTTTCTCCATACCCGTTTTTTTGGGCTAGCAGGAGGATTACACCATGGACGATTTAA CCCTGCGTTATTTTGACGCTG
BAD33-TssF-FLAG-3	GGTCGACTCTAGAGGATCCCCGGGTACCTTA**TTTATCATCGTCGTCTTTATAATC**ACCGGGCAG GCGCGGATTGTG
BAD33-TssG-5	CTCTCTACTGTTTCTCCATACCCGTTTTTTTGGGCTAGCAGGAGGATTACACCATGTTGCTGGAAC AAAGCCAGCCAGACCAG
BAD33-TssG-FLAG-3	GGTCGACTCTAGAGGATCCCCGGGTACCTTA**TTTATCATCGTCGTCTTTATAATC**CCAGCGATA ATCGCCATTTTCCTGAGTCTCC
BAD33-TssG-VSVG-3	GGTCGACTCTAGAGGATCCCCGGGTACCTTA**TTTTCCTAATCTATTCATTTCAATATCTGTATA** CCAGCGATAATCGCCATTTTCCTG
BAD33-VgrG-ΔOB-VSVG-5	CTCTCTACTGTTTCTCCATACCCGTTTTTTTGGGCTAGCAGGAGGTATTACACCATGAATCTCACT GACTCCCTGCAAAATGTTTTATCC
BAD33-VgrG-ΔOB-VSVG-3	GGTCGACTCTAGAGGATCCCCGGGTACCTTA**TTTTCCTAATCTATTCATTTCAATATCTGTATA** CTTTCCTTGTGCCTGAGGCTGCATATC

BAD33-VgrG-VSVG-3	GGTCGACTCTAGAGGATCCCCGGGTACCTTA**TTTTCCTAATCTATTCATTTCAATATCTGTATA**TTCTGTTTCTCCATGAATTTTTACCTTCCCAAACTC
BAD33-TssA-5	CTCTCTACTGTTTCTCCATACCCGTTTTTTTGGGCTAGCAGGAGGTATTACACCATGGCTTCCATTCATTCGCTCCTCAGTG
BAD33-TssA-VSVG-3	GGTCGACTCTAGAGGATCCCCGGGTACCTTA**TTTTCCTAATCTATTCATTTCAATATCTGTATA**TGAACTTCCCGAATTGCACAAC
BAD33-TssE-5	CTCTCTACTGTTTCTCCATACCCGTTTTTTTGGGCTAGCAGGAGGTATTACACCATGGTGATGCCGCGTCCTTCCCTTTATG
BAD33-TssE-VSVG-3	GGTCGACTCTAGAGGATCCCCGGGTACCTTA**TTTTCCTAATCTATTCATTTCAATATCTGTATA**CGTCTGCACGTAGCGCTGCTG
BAD33-TssK-5	CTCTCTACTGTTTCTCCATACCCGTTTTTTTGGGCTAGCAGGAGGTATTACACCATGAAGATTTATCGCCCATTATGGGAAGACGGG
BAD33-TssK-VSVG-3	GGTCGACTCTAGAGGATCCCCGGGTACC**TTATTTTCCTAATCTATTCATTTCAATATCTGTATA**TGTCCGCAGCACCGCAAAAAGTTC
T25T18C-5-TssF+G	CGGATAACAATTTCACACAGGAAACAGCTATGACCATGGACGATTTAACCCTGCGTTATTTGACG
T25C-3- TssF+G	GTTTGCGTAACCAGCCTGATGCGATTGCTGCCAGCGATAATCGCCATTTTCCTGAGTCTC
T18C-3- TssF+G	CCTCGCTGGCGGCTAAGCTTGGCGTAATCCAGCGATAATCGCCATTTTCCTGAGTCTC
T25T18N-3-TssF+G	CGAGGTCGACGGTATCGATAAGCTTGATATCGAATTCTAGTTAACCGGGCAGGCGCGGATTGTG
T25N-5- TssF+G	GGCGGGCTGCAGATTATAAAGATGACGATGACAAGTTGCTGGAACAAAGCCAGCCAGACCAG
T18N-5- TssF+G	CGCCACTGCAGGGATTATAAAGATGACGATGACAAGTTGCTGGAACAAAGCCAGCCAGACCAG
pKD4-*gfp*-5	CGGAATAGGAACTAAGGAGGATATTCATATGTCTAAAGGTGAAGAACTGTTCACCG
pKD4-*gfp*-3	CTGACATGGGAATTAGCCATGGTCCCCTCCGCCGGCCGCTGCTTTGTAGAGCTCATCCATGCCG
p*m-Ch*-KD4-5	GCAGCATTACACGTCTTGAGCGATTGCAGCGGCCGGCGGAGG
p*m-Ch*-KD4-3	CTTCGAAGCAGCTCCAGCCTACACTTACTTGTACAGCTCGTCCATGCCGCC

[a] Sequences corresponding to the downstream and upstream regions of the gene to be deleted underlined.
[b] Sequence annealing on the target gene underlined.
[c] Restriction site *italicized*.
[d] FLAG or VSV-G epitope coding sequence in **Bold.**

References

Aschtgen MS, Bernard CS, De Bentzmann S, Lloubès R, Cascales E (2008) SciN is an outer membrane lipoprotein required for type VI secretion in enteroaggregative Escherichia coli. J Bacteriol. 190: 7523-31.

Aschtgen MS, Gavioli M, Dessen A, Lloubès R, Cascales E (2010) The SciZ protein anchors the enteroaggregative *Escherichia coli* Type VI secretion system to the cell wall. Mol Microbiol. 75: 886-99.

Battesti A, Bouveret E (2008) Improvement of bacterial two-hybrid vectors for detection of fusion proteins and transfer to pBAD-tandem affinity purification, calmodulin binding peptide, or 6-histidine tag vectors. Proteomics. 8: 4768-71.

Brunet YR, Hénin J, Celia H, Cascales E (2014) Type VI secretion and bacteriophage tail tubes share a common assembly pathway. EMBO Rep. 15: 315-21.

Datsenko KA, Wanner BL (2000) One-step inactivation of chromosomal genes in *Escherichia coli* K-12 using PCR products. Proc Natl Acad Sci USA. 97: 6640-5.

Durand E, Nguyen VS, Zoued A, Logger L., Pehau-Arnaudet G., Aschtgen MS, Spinelli S, Desmyter A, Bardiaux B, Dujeancourt A, Roussel A, Cambillau C, Cascales E, Fronzes R (2015) Biogenesis and structure of a Type VI secretion membrane core complex. *Nature*. in press. doi :10.1038/nature14667.

Durand E, Zoued A, Spinelli S, Watson PJ, Aschtgen MS, Journet L, Cambillau C, Cascales E (2012) Structural characterization and oligomerization of the TssL protein, a component shared by bacterial type VI and type IVb secretion systems. J Biol Chem. 287: 14157-68.

Guzman LM, Belin D, Carson MJ, Beckwith J (1995) Tight regulation, modulation, and high-level expression by vectors containing the arabinose PBAD promoter. J Bacteriol. 177: 4121-30.

Karimova G, Pidoux J, Ullmann A, Ladant D (1998) A bacterial two-hybrid system based on a reconstituted signal transduction pathway. Proc Natl Acad Sci USA. 95: 5752-6.

Vieira J, Messing J (1991) New pUC-derived cloning vectors with different selectable markers and DNA replication origins. Gene. 100: 189-94.

Zoued A, Durand E, Bebeacua C, Brunet YR, Douzi B, Cambillau C, Cascales E, Journet L (2013) TssK is a trimeric cytoplasmic protein interacting with components of both phage-like and membrane anchoring complexes of the type VI secretion system. J Biol Chem. 288: 27031-41.
