# Supplementary figures and images for: The Type VI Secretion TssEFGK-VgrG Phage-Like Baseplate Is Recruited to the TssJLM Membrane Complex via Multiple Contacts and Serves As Assembly Platform for Tail Tube/Sheath Polymerization
Source: PLoS Genet. 2015 Oct 13;11(10):e1005545. doi: 10.1371/journal.pgen.1005545 (PMC4604203; doi:10.1371/journal.pgen.1005545)

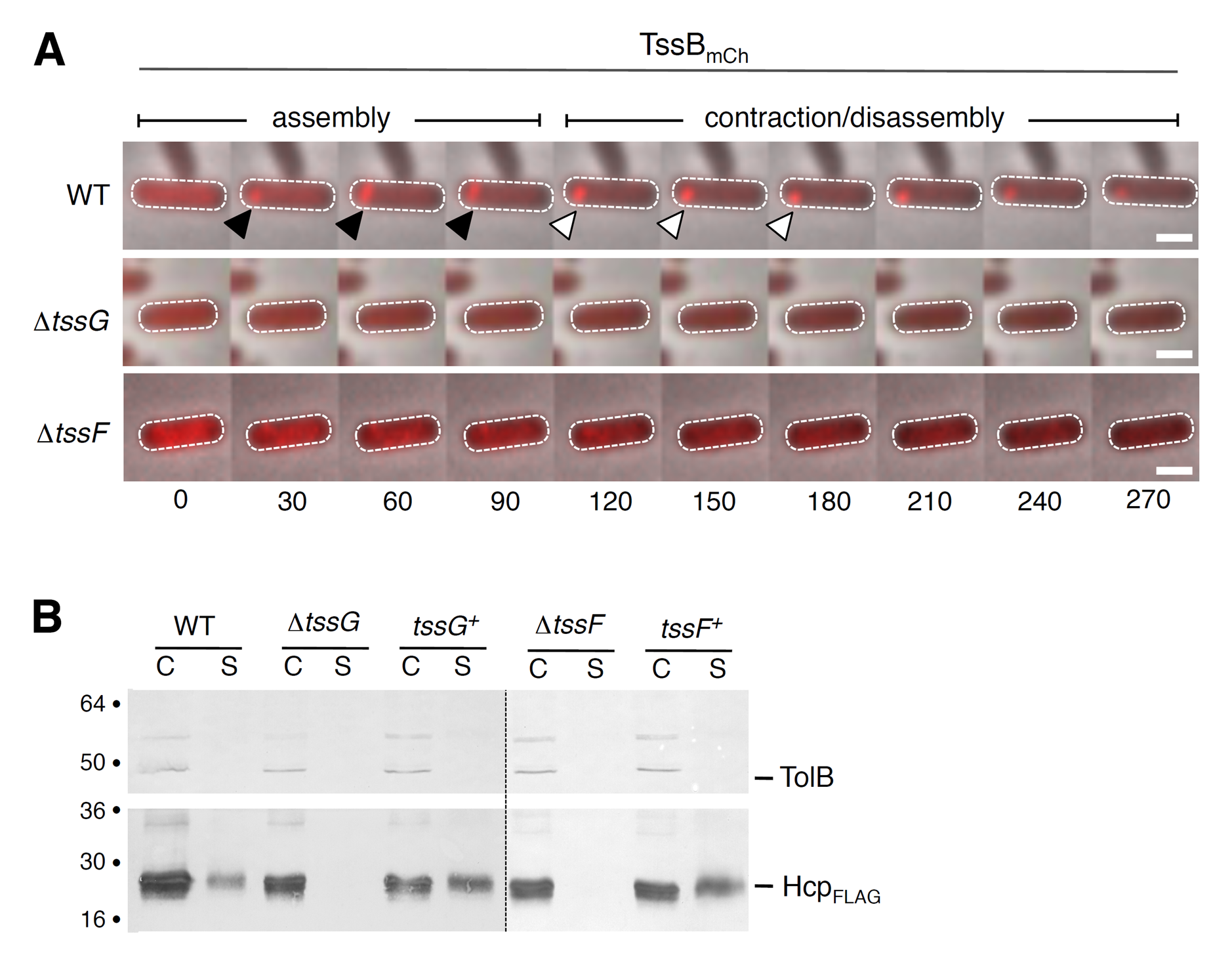

Supplement: S1 Fig — Effect of the tssF and tssG mutations on T6SS sheath formation (A) and Hcp protein release (B). (A) Time-lapse fluorescence recordings of WT, tssF or tssG cells carrying the tssB-mCherry chromosomal fusion at the original locus. Individual images were taken every 30 sec. Assembly and contraction/disassembly events are indicated by the black and white triangles respectively. Scale bars are 1 m. (B) HcpFLAG release was assessed by separating whole cells (C) and supernatant (S) fractions from WT, tssF or tssG, and complemented tssF or tssG (tssF + or tssG +) cultures. A total of 2×10^8^ cells and the TCA-precipitated material of the supernatant from 5×10^8^ cells were loaded on a 12.5%-acrylamide SDS-PAGE and immunodetected using the anti-FLAG monoclonal antibody (lower panel) and the anti-TolB polyclonal antibodies (control for cell integrity; upper panel). (TIF) [file pgen.1005545.s002.tif]

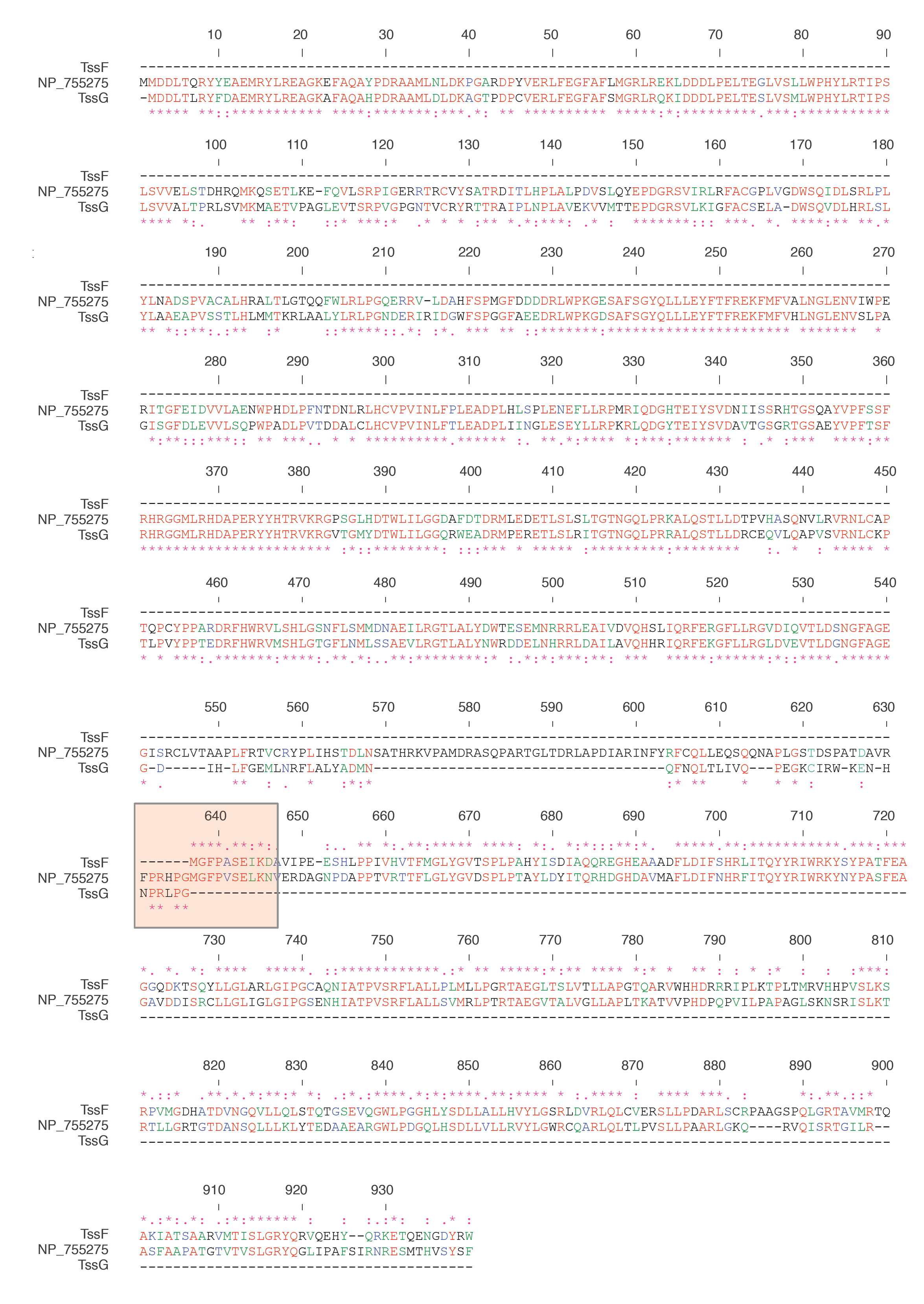

Supplement: S2 Fig — TssF and the N-terminal region of NP_7555275 share 58% of identity and 75% of similarity. TssG and the C-terminal region of NP_7555275 share 53% of identity and 82% of similarity. The region corresponding to the fusion is framed. (TIF) [file pgen.1005545.s003.tif]

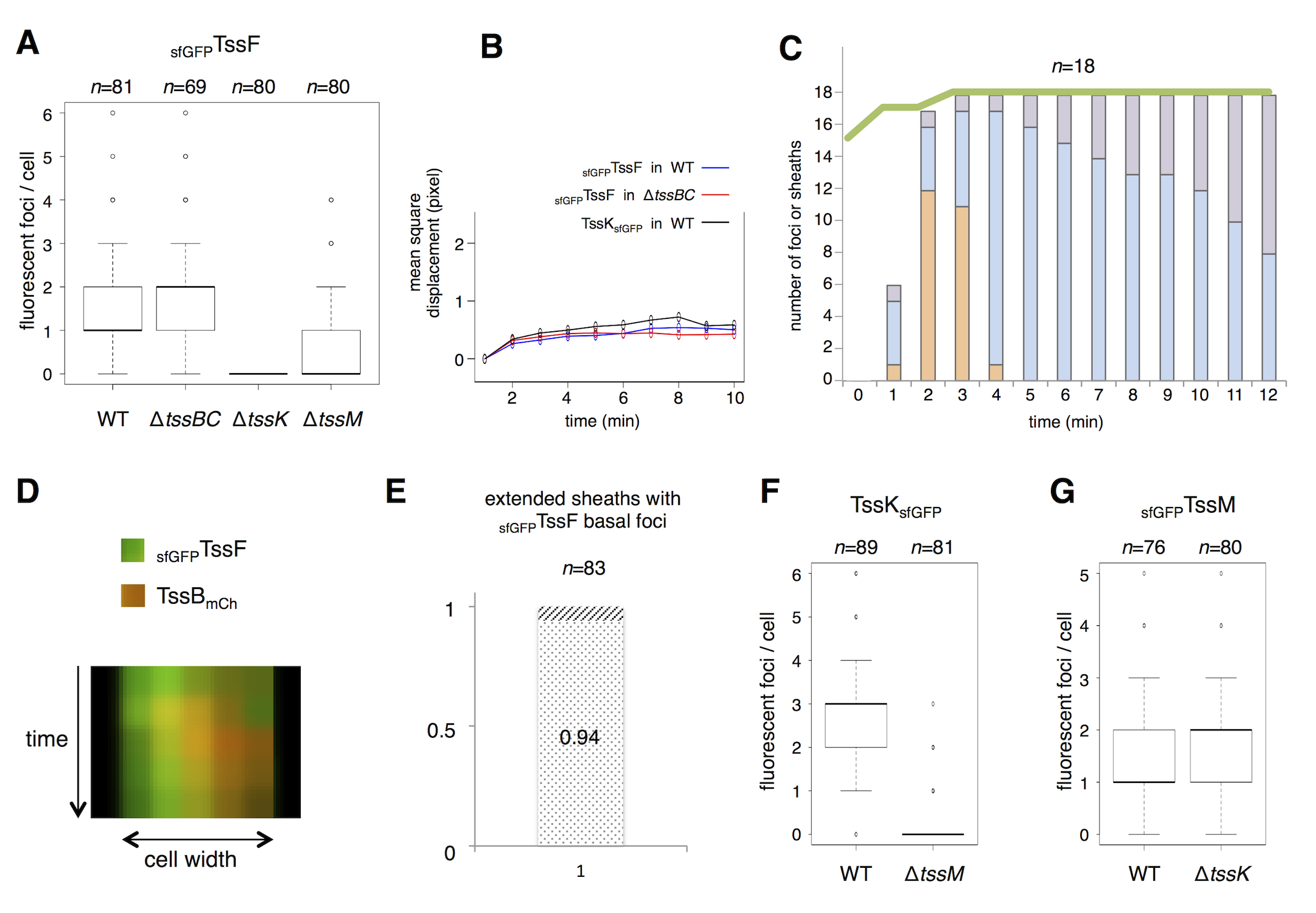

Supplement: S3 Fig — Statistical analyses of sfGFPTssF (A), TssKsfGFP (F) or sfGFPTssM (G) localization in the indicated strains. Shown are box-and-whisker plots of the measured number of sfGFPTssF, TssK sfGFP or sfGFPTssM foci per cell for each strain with the lower and upper boundaries of the boxes corresponding to the 25% and 75% percentiles respectively. The black bold horizontal bar represents the median values for each strain and the whiskers represent the 10% and 90% percentiles. Outliers are shown as open circle. n indicates the number of cells analyzed per strain. (B) sfGFPTssF and TssKsfGFP foci are stable and static. Mean square displacement (in pixel) of sfGFPTssF clusters in WT (blue graph) or ΔtssBC cells (red graph) and TssKsfGFP clusters in WT cells (black graph) were measured by sub-pixel tracking of fluorescent foci and plotted over time (in minutes). (C) sfGFPTssF clusters assemble prior to TssBC sheaths. Kinetics of apparition of sfGFPTssF clusters (green line) and dynamics of TssBmCh sheaths (bars; orange: elongation, blue: elongated; purple: contraction/disassembly) plotted over time (in minutes). (D) sfGFPTssF remains at the base of the sheath during elongation. Kymographic analysis reporting sfGFPTssF (green) and TssBmCh (red) positions within the cell as a function of time. (E) Percentage of sheaths (identified as elongated TssBmCh structures) with basal sfGFPTssF clusters (identified by GFP-labeled foci), (dotted bar) in the strain producing both sfGFPTssF and TssBmCh. n indicates the number of cells analyzed. (TIFF) [file pgen.1005545.s004.tiff]

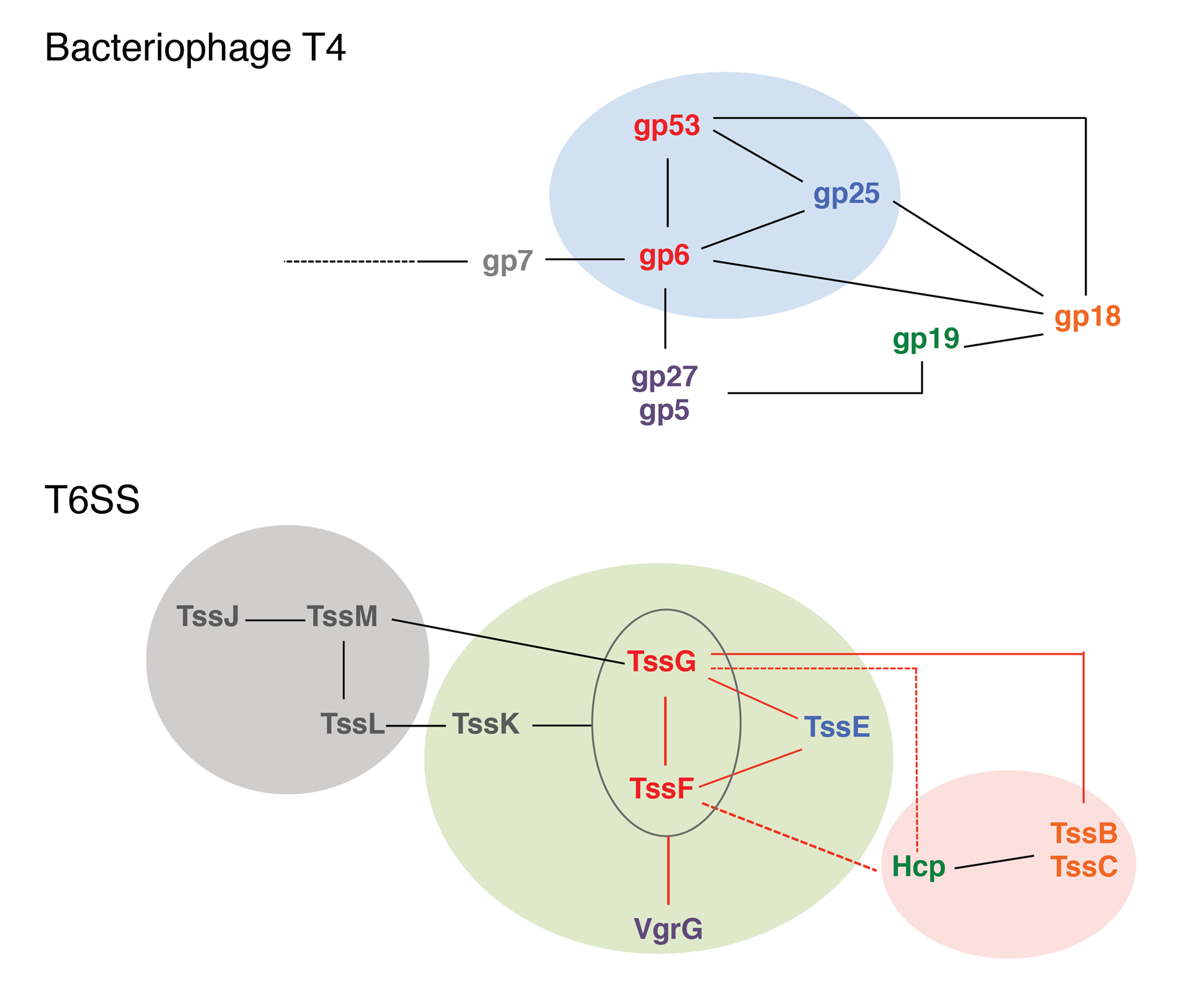

Supplement: S4 Fig — Schematic representation of known interactions between selected bacteriophage T4 components (top) or between T6SS components (bottom). Proteins sharing sequence or structural homologies are indicated with the same color. The bacteriophage T4 wedge components are indicated in the blue box, while the T6SS membrane complex (MC), baseplate complex (BC) and tail complex (TC) are boxed in grey, green and red respectively. (TIF) [file pgen.1005545.s005.tif]
